# Infinitesimal Jackknife Estimates of Standard Errors for Rotated Estimates of Redundancy Analysis: Applications to Two Real Examples

**DOI:** 10.1017/psy.2024.8

**Published:** 2025-01-03

**Authors:** Fei Gu, Somboon Jarukasemthawee, Kullaya Pisitsungkagarn, Ynte K. van Dam

**Affiliations:** 1 Faculty of Psychology, Chulalongkorn University, Bangkok, Thailand; 2 Research Unit on Disaster Psychology and Well-being, Chulalongkorn University, Bangkok, Thailand; 3 Marketing and Consumer Behaviour Group, Wageningen University, Wageningen, The Netherlands

**Keywords:** redundancy analysis, rotated estimates, standard error estimates, infinitesimal jackknife

## Abstract

In redundancy analysis (RA), the redundancy variates are interpreted in terms of the predictor variables that have the prominent redundancy loadings. Israels (1986) advocated the rotation of redundancy loadings to facilitate the interpretation of the rotated redundancy variates. In this paper, the purpose is to obtain the standard error estimates for rotated redundancy loadings that can facilitate the interpretation of the rotated redundancy variates. To this end, we modify the original RA-L model (Gu et al., 2023) and specify two modified RA-L models for orthogonal and oblique rotations, separately. On the basis of the modified RA-L models, we describe the infinitesimal jackknife (IJ) method that can produce the standard error estimates for rotated RA estimates. A simulation study is conducted to validate the standard error estimates from the IJ method, and two real examples are used to demonstrate the use of the standard error estimates for rotated redundancy loadings. Finally, we summarize the paper and provide additional remarks regarding the rotation methods and the use of numeric derivatives in the implementation of the IJ method.

## 1 Introduction

Canonical correlation analysis (CCA; Hotelling, 1935, 1936) and redundancy analysis (RA; Van Den Wollenberg, 1977) are two classic multivariate statistical methods that can be used to study the relationship between two sets of variables. In CCA, the first pair of canonical variates (i.e., linear combinations of original variables) is created from both sets to maximize the first canonical correlation (i.e., the correlation between the paired canonical variates), and subsequent pairs of canonical variates are created to maximize the following canonical correlations while obeying certain within-set and between-set orthogonality restrictions. One potential disadvantage of CCA is that the canonical variates may not be representative of the original variables in the sense of the explained variance within the same set. For instance, if all the canonical variates created from the first set can only explain 5% (or even less) of the variance of the original variables in the first set and all the canonical variates created from the second set can only explain 5% (or even less) of the variance of the original variables in the second set, no matter how large the canonical correlations are, it is impossible to have a big overlap in variance between the two sets of original variables (Fornell, 1979; Van Den Wollenberg, 1977). As a remedy, RA was proposed to create the redundancy variates (i.e., linear combinations of original variables) from only one set of original variables (say, the predictor variables) with the goal of maximizing the explained variance of the other set of original variables (say, the criterion variables). Mathematically, the redundancy variates can also be created from the criterion variables to maximize the explained variance of the predictor variables, but it is often not necessary to do so for theoretical reasons.

Despite the differences in mathematical goals, the two methods are similar in the sense that the interpretations of the linear combinations of original variables are often the focus of practical applications of the two methods. To interpret the canonical variates in CCA, researchers should select the original variables with prominent canonical loadings (i.e., the correlations between the canonical variates and the original variables within the same set) to assign meaningful interpretation to each canonical variate. In a similar way, a redundancy variate should be interpreted in terms of the predictor variables with prominent redundancy loadings (i.e., the correlations between the redundancy variates and the predictor variables). Nonetheless, there is no guarantee that meaningful interpretations can always be found for the canonical/redundancy variates.

To facilitate the interpretations, the idea of rotation that was originally developed to rotate the common factors in the context of exploratory factor analysis (EFA) has been adapted to rotate the canonical/redundancy variates. In the CCA context, Cliff and Krus (1976) and Perreault and Spiro (1978) advocated the rotation of canonical variates, whereas, in the RA context, Israels (1986) discussed the rotation of redundancy variates. These authors showed that the rotated canonical/redundancy loading matrix often has a simple structure in the sense of Thurstone (1947), which makes it easier to interpret the rotated canonical/redundancy variates. Additionally, Cudeck and O’Dell (1994) suggested the use of standard error estimates to account for the sampling variability of rotated factor loadings when the rotated common factors are interpreted. Following this suggestion, Gu et al. (2021) developed the standard error estimates for rotated canonical loadings and other rotated CCA estimates. However, no work has been done to obtain the standard error estimates for rotated redundancy loadings or other rotated RA estimates. Therefore, the purpose of this paper is to develop the standard error estimates for rotated RA estimates. With the availability of standard error estimates, the researcher can better interpret the rotated redundancy variates by selecting the rotated redundancy loadings that are not only prominent but also statistically significant.

Because the technical details in this paper are closely related to Gu et al. (2021), it is useful to review the related work that leads to the standard error estimates for rotated CCA estimates. It is well known that CCA is almost always used in exploratory data analysis, because the traditional development of CCA does not provide the inferential information to test the CCA parameters, except the canonical correlations, of which the significance can be tested under the multivariate normality assumption of the data. Recently, Gu et al. (2019) provided a model-based approach to CCA that can produce the standard error estimates for CCA estimates. Particularly, their model-based approach includes four covariance structure models^1^ specifically designed for CCA, and one of the models (i.e., the CORR-L model) can produce the standard error estimates for canonical loadings. Based on the original CORR-L model, Gu et al. (2021) provided the specification of the modified CORR-L model that can accommodate the rotated canonical loadings and other rotated CCA estimates; and they further showed that the infinitesimal jackknife (IJ) method^2^ (Jennrich & Clarkson, 1980; Jennrich, 2008; Zhang et al., 2012) can be applied with the modified CORR-L model to compute the standard error estimates for rotated canonical loadings and other rotated CCA estimates. The advantage of the IJ method is that it can handle non-normal data and produce robust standard error estimates. Thus, we also focus on the IJ method in this paper. In sum, it is the modified CORR-L model that serves as the basis for applying the IJ method.

Based on the work of Gu et al. (2021) in the CCA context, we can easily outline the work required to produce the standard error estimates for rotated redundancy loadings and other rotated RA estimates. First, we need a model that can accommodate the rotated RA estimates. Then, we can apply the IJ method with the specified model to compute the standard error estimates for rotated RA estimates. Recently, Gu et al. (2023) developed a model-based approach to RA that can produce the standard error estimates for RA estimates. Particularly, their model-based approach includes two covariance structure models^3^ specifically designed for RA, and one of the models (i.e., the RA-L model) can produce the standard error estimates for redundancy loadings. Thus, a feasible way to develop a model that can accommodate the rotated redundancy loadings and other rotated RA estimates is to modify the original RA-L model. Then, the IJ method can be applied with the modified RA-L model. Hence, the required work is to specify the modified RA-L model, because the modified RA-L model serves as the basis to apply the IJ method to compute the standard error estimates for rotated RA estimates.

The organization of this paper is as follows. In Section 2, we first review the original RA-L model; then, we specify two modified RA-L models to accommodate the rotated RA estimates from orthogonal and oblique rotations, separately. In Section 3, we describe the IJ method with the two modified RA-L models estimated by the unweighted least squares (ULS) fitting function. In Section 4, we use a simulation study to validate the standard error estimates from the IJ method. In Section 5, we use two real examples to demonstrate the interpretation of rotated redundancy variates. Finally, in Section 6, we summarize the paper and provide additional remarks regarding the rotation methods and the use of numeric partial derivatives when applying the IJ method.

## 2 The original RA-L model and two modified RA-L models

In this section, we first review the original RA-L model and then specify two modified RA-L models for orthogonal and oblique rotations, separately.

### 2.1 The original RA-L model

Let **x** be a *p* × 1 vector for *p* predictor variables and **y** be a *q* × 1 vector for *q* criterion variables. With *p* predictor variables, one can construct up to *p* redundancy variates. Let 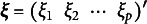 be the vector that includes all *p* redundancy variates. According to Van Den Wollenberg (1977), *ξ_i_* (*i* = 1, 2, …, *p*) must satisfy two restrictions. First, *ξ_i_* is uncorrelated with *ξ_j_* (*i* ≠ *j*). Second, *ξ_i_* has unit variance (*i* = 1, 2, …, *p*). With these restrictions, Gu et al. (2023) specified the covariance structure of the original RA-L model as(1)

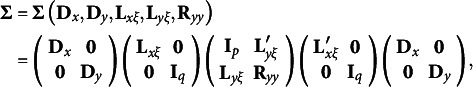

 where **I** *_p_* and **I** *_q_* are identity matrices of orders *p* and *q*, separately, **D** *_x_* is a *p* × *p* diagonal matrix whose diagonal elements are the standard deviations of *p* predictor variables, **D** *_y_* is a *q* × *q* diagonal matrix whose diagonal elements are the standard deviations of *q* criterion variables, **L** *_xξ_* is a *p* × *p* square matrix that includes the redundancy loadings (i.e., the correlations between *p* predictor variables and *p* redundancy variates), **L** *_yξ_* is a *q* × *p* matrix that includes the cross-loadings (i.e., the correlations between *q* criterion variables and *p* redundancy variates), and **R** *_yy_* is a *q* × *q* correlation matrix whose off-diagonal elements are the correlations of *q* criterion variables.

To identify the original RA-L model, three types of constraints must be imposed. The first type of constraints is applicable only when the number of predictor variables exceeds that of criterion variables by two or more (i.e., *p* - *q* ≥ 2). Specifically, let *d* = *p* − *q* be a positive integer. When *d* ≥ 2, the first type of constraints requires one to arbitrarily fix *d*(*d* − 1)/2 elements in the last *d* columns of **L** *_xξ_*. When *d* = 1 or *p* ≤ *q*, the first type of constraints is not applicable. The second type of constraints is(2)

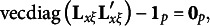

 where vecdiag(**M**) denotes a column vector created with the diagonal elements of **M**, and **1** *_p_* denotes a unit vector of order *p*, and **0** *_p_* denotes a null vector of order *p*. Finally, the third type of constraints is(3)

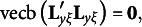

 where vecb(**M**) denotes a column vector created with the off-diagonal elements below the main diagonal of **M**, and **0** denotes a null vector of appropriate order^4^. The third type of constraints indicate that 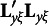 must be a diagonal matrix, but the number of constraints required by equation (3) depends on the relative magnitude of *p* and *q*. When *p* ≤ *q*, all *p* columns of **L** *_yξ_* include non-zero cross-loadings. In this situation, 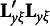 has *p*(*p* − 1)/2 unique off-diagonal elements that must be 0. When *p* > *q*, only the first *q* columns of **L** *_yξ_* include non-zero cross-loadings, while the last *d* = *p* - *q* columns of **L** *_yξ_* are null vectors (see Appendix A of Gu et al. 2023). In this situation, the first *q* × *q* submatrix of 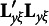 has *q*(*q* − 1)/2 unique off-diagonal elements that must be 0. This completes the three types of constraints for the original RA-L model.

To count the number of parameters of the RA-L model, it is obvious that **D** *_x_* has *p* standard deviations, **D** *_y_* has *q* standard deviations, and **R** *_yy_* has *q*(*q* − 1)/2 correlations. For **L** *_xξ_* and **L** *_yξ_*, however, the number of parameters in these two matrices also depends on the relative magnitude of *p* and *q*. For *p* ≤ *q*, **L** *_xξ_* has *p*^2^ redundancy loadings, and **L** *_yξ_* has *pq* cross-loadings. For *p* > *q*, **L** *_xξ_* has *p*^2^ − *d*(*d* − 1)/2 = (*p*^2^ + 2*pq* − *q*^2^ + *p* − *q*)/2 redundancy loadings, and **L** *_yξ_* has *q*^2^ cross-loadings in the first *q* columns because the last *d* columns of **L** *_yξ_* are null vectors. Finally, given the number of constraints for identification and the number of parameters, we can verify that the RA-L model is a saturated model regardless of the relative magnitude of *p* and *q* (see Appendix B of Gu et al. 2023).

### 2.2 Matrix partitions

To specify the two modified RA-L models in the next two subsections, it is necessary to partition some matrices of the original RA-L model. Let *m* be a positive integer that indicates the number of redundancy variates to be rotated. When *p* ≤ *q*, *m* must be equal to or less than *p*. When *p* > *q*, *m* must be equal to or less than *q*, because there is no need to rotate the last *d* = *p* − *q* redundancy variates.

With these settings, we first partition **L** *_xξ_* as(4)

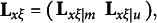

 where 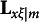 is a *p* × *m* matrix, 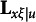 is a *p* × *u* matrix, and *u* = *p* - *m*. Correspondingly, the submatrices **I** *_p_* and **L** *_yξ_* in 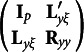 of equation (1) should be partitioned as(5)

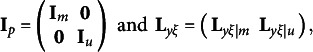

 where 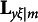 is a *q* × *m* matrix and 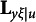 is a *q* × *u* matrix.

Based on the partitions in equations (4) and (5), the covariance structure of the original RA-L model can be re-written as(6)

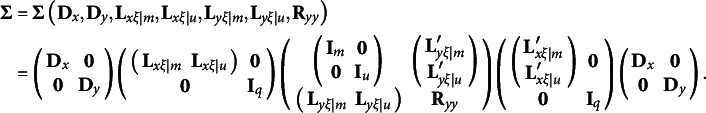

In the next two subsections, we will show the effect of orthogonal and oblique rotations on 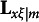 , **I** *_m_*, and 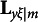 in equation (6) and define the two modified RA-L models for orthogonal and oblique rotations, separately.

### 2.3 The modified RA-L model for orthogonal rotations

When the first *m* redundancy variates are rotated with an orthogonal rotation method, 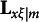 is transformed by an *m* × *m* orthogonal matrix **T**^orth^ to produce 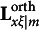 , which is a *p* × *m* matrix that includes the rotated redundancy loadings. That is,(7)

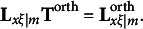

At the same time, **I** *_m_* and 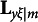 are also transformed by **T**^orth^. For **I** *_m_*, the transformation is(8)

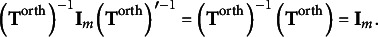

For 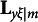 , the transformation is(9)

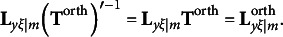

Obviously, 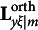 is a *q* × *m* matrix that includes the rotated cross-loadings. Given equations (7)–(9), the covariance structure of the modified RA-L model for orthogonal rotations is defined as(10)

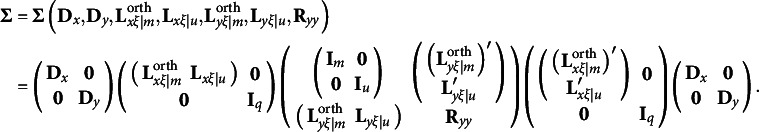



To identify the modified RA-L model for orthogonal rotations, we must impose four types of constraints. The first three types of constraints are inherited with or without changes from the three types of constraints for the original RA-L model, whereas the fourth type of constraints is introduced to remove rotational indeterminacy. The first type of constraints is identical to that for the original RA-L model. That is, when **x** has 2 or more variables than **y**, one should arbitrarily fix *d*(*d* − 1)/2 elements in the last *d* columns of 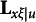 .

The second type of constraints involves both rotated and unrotated redundancy loadings. That is,(11)

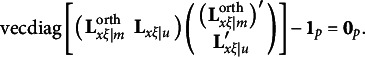

Compared to the *p* constraints in equation (2), the first *m* constraints in equation (11) are different, because these constraints are imposed on the rotated redundancy loadings in 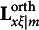 .

To derive the third type of constraints, we must express 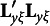 in equation (3) with the partitioned matrix 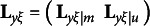 . That is,

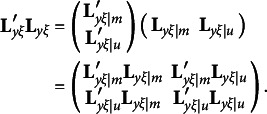



Given the constraints required by equation (3), we can see that 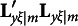 and 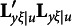 must be diagonal matrices and 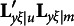 must be a null matrix. Thus, we can re-write equation (3) as

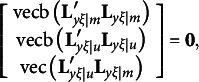

 where vec(**M**) denotes a column vector created with all elements of **M**. With orthogonal rotations, 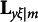 should be substituted with 
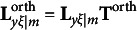
 so that the first and last components in the above expression must be changed as follows:

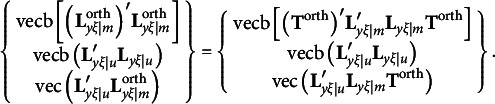

It is easy to verify that 
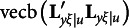
 and 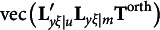 remain to be null vectors after orthogonal rotations, but 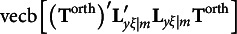 may not be a null vector, because 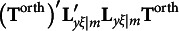 in general is an *m* × *m* symmetric matrix. It means that rotation violates the first *m*(*m* − 1)/2 constraints required by equation (3). Therefore, the third type of constraints for the modified RA-L model for orthogonal rotations is(12)

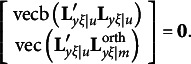



In the fourth type of constraints, the results derived by Archer and Jennrich (1973) are adapted to remove rotational indeterminacy for orthogonal rotations. That is, the fourth type of constraints requires 
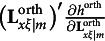
 to be a symmetric matrix, where 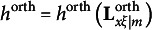 denotes the simplicity function of 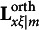 for a particular orthogonal rotation criterion, and this type of constraints includes *m*(*m* − 1)/2 constraints. Formally, we can write the fourth type of constraints as(13)

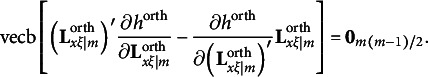

This completes the four types of constraints for the modified RA-L model for orthogonal rotations.

It can be seen that the number of parameters of the modified RA-L model for orthogonal rotations is the same as that of the original RA-L model, because orthogonal rotations do not increase the number of parameters. As for the number of constraints, equation (12) has *m*(*m* − 1)/2 fewer constraints than equation (3), while equation (13) introduces *m*(*m* − 1)/2 new constraints. Therefore, the modified RA-L model for orthogonal rotations is still a saturated model.

### 2.4 The modified RA-L model for oblique rotations

When the first *m* redundancy variates are rotated with an oblique rotation method, 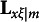 is transformed by an *m* × *m* nonsingular matrix **T**^obli^ that must satisfy the restriction 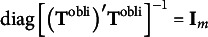 to produce 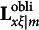 , which is a *p* × *m* matrix that includes the rotated redundancy loadings. That is,(14)

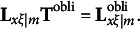

At the same time, **I** *_m_* and 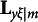 are also transformed by **T**^obli^. For **I** *_m_*, the transformation is(15)

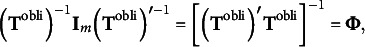

 where is **Φ** a *m* × *m* correlation matrix^5^ of the rotated redundancy variates. For 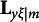 , the transformation is(16)

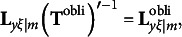

 where 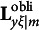 is a *q* × *m* matrix that includes the rotated cross-loadings. Based on equations (14)–(16), the covariance structure of the modified RA-L model for oblique rotations is defined as(17)

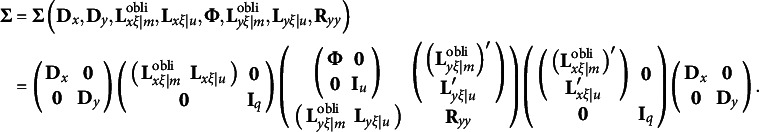

Note that equation (17) has *m*(*m* − 1)/2 more parameters than equations (6) due to the off-diagonal elements of **Φ**.

To identify the modified RA-L model for oblique rotations, we also need to impose four types of constraints. The first type of constraints is that when **x** has 2 or more variables than **y**, one should arbitrarily fix *d*(*d* − 1)/2 elements in the last *d* columns of 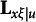 in equation (17).

The second type of constraints involves not only the rotated and unrotated redundancy loadings but also the correlations of the rotated redundancy variates. That is,(18)

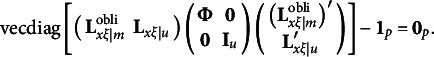

Compared to the *p* constraints in equation (2), the first *m* constraints in equation (18) are different, because these *m* constraints involve the rotated redundancy loadings in 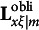 and the correlations in **Φ**.

The derivation of the third type of constraints for the modified RA-L model for oblique rotations is similar to that for the orthogonal rotations. Recall that equation (3) requires

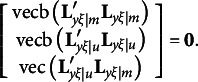

With oblique rotations, 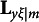 should be substituted with 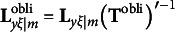 so that the first and last components in the above expression must be changed as follows:

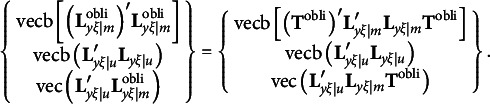

It is easy to verify that 
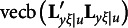
 and 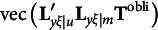 remain to be null vectors after oblique rotations, but 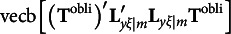 may not be a null vector, because 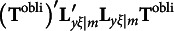 in general is an *m* × *m* symmetric matrix. Therefore, the third type of constraints for the modified RA-L model for oblique rotations is(19)

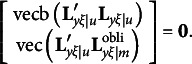



In the fourth type of constraints, the results derived by Jennrich (1973) are adapted to remove rotational indeterminacy for oblique rotations. That is, the fourth type of constraints requires 
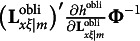
 to be a diagonal matrix, where 
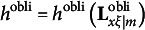
 denotes the simplicity function of 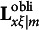 for a particular oblique rotation criterion, and this type of constraints includes *m*(*m* − 1) constraints. Formally, we can write the fourth type of constraints as(20)

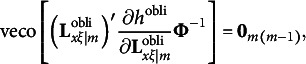

 where veco(**M**) denotes a column vector created with all off-diagonal elements of **M**. This completes the four types of constraints for the modified RA-L model for oblique rotations.

It can be seen that the modified RA-L model for oblique rotations has *m*(*m* − 1)/2 more parameters (i.e., the off-diagonal elements of **Φ**) than the original RA-L model, equation (19) has *m*(*m* − 1)/2 fewer constraints than equation (3), and equation (20) introduces *m*(*m* − 1) new constraints. Therefore, the modified RA-L model for oblique rotations is still a saturated model.

## 3 The infinitesimal jackknife method

In this section, we describe the IJ method with the modified RA-L models estimated by the ULS fitting function. Computationally, the IJ method requires the pseudo values, which are obtained from two quantities: 1) the Jacobian matrix of the estimating equations with respect to the estimates and 2) the partial differentials of the estimating equations with respect to the sample covariance matrix **S**. The Jacobian matrix and the partial differentials are described first, followed by the descriptions of the pseudo values and the IJ estimate of the asymptotic covariance matrix.

### 3.1 Notations of the parameter vectors

Strictly speaking, we should use **θ**^orth^ and **θ**^obli^ to denote the parameter vectors for the two modified RA-L models, separately. With these notations, we have 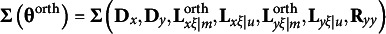 and 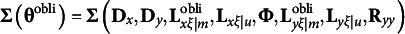 . However, to avoid repetitive descriptions in this section, we use **θ** as a generic symbol to denote the parameter vector for both modified RA-L models. As such, 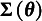 is used to refer to either 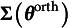 or 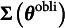 .

### 3.2 Jacobian matrix and partial differentials

For both modified RA-L models, the ULS fitting function is defined as(21)

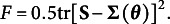



Then, the estimating equations have the following form(22)

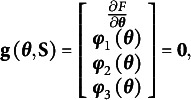

 where 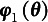 , 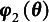 , and 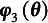 represent the second, third, and fourth type of constraints for either modified RA-L model. Specifically, 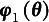 includes *p* constraints from either equation (11) for orthogonal rotations or equation (18) for oblique rotations, 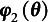 includes *p*(*p* − 1)/2 − *m*(*m* − 1)/2 or *q*(*q* − 1)/2 − *m*(*m* − 1)/2 constraints, depending on the relative magnitude of *p* and *q*, from either equation (12) for orthogonal rotations or equation (19) for oblique rotations, and 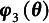 includes either *m*(*m* − 1)/2 constraints from equation (13) for orthogonal rotations or *m*(*m* − 1) constraints from equation (20) for oblique rotations.

Given equation (22), the Jacobian matrix of 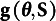 with respect to **θ** is(23)

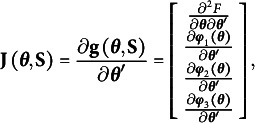

 where 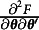 is the Hessian matrix of the ULS fitting function, and the remaining components are the partial derivatives of the constraints with respect to **θ**.

Let 
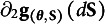
 be the partial differential of 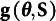 with respect to **S** evaluated at 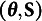 , and we define **k** *_n_* as(24)

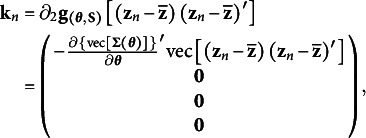

 where *n* = 1, 2, …, *N*, *N* is the sample size, **z** *_n_* is a column vector for the *n*th observation of all predictor and criterion variables, and 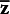 is a column vector of the sample means of all predictor and criterion variables. The last three components in equation (24) are null vectors, because 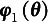 , 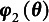 , and 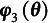 are not functions of **S**.

### 3.3 Pseudo values and asymptotic covariance matrix of parameter estimates

Given the Jacobian matrix and the partial differentials, the pseudo values for each observation can be computed. Let **λ** *_n_* (*n* = 1, …, *N*) be a column vector collecting the pseudo values for the *n*th observation, and it can be solved from(25)

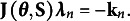

Note that 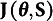 defined in equation (23) has more rows than columns so that the system of equations in equation (25) appears to be over-determined. Thus, we apply the QR decomposition to 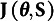 to solve for **λ** *_n_*.

After **λ** *_n_* is obtained for all observations, the IJ estimate of the asymptotic covariance matrix of 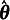 is(26)

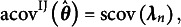

 where 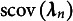 is the sample covariance matrix of all **λ** *_n_*. Finally, the standard error estimates for 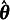 are obtained from dividing the square roots of the diagonal elements of 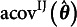 by 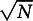 .

## 4 A simulation study

In this section, we use a simulation study to validate the standard error estimates from the IJ method under both multivariate normality and multivariate nonnormality and at different sample sizes.

### 4.1 Data generation

Two factors are manipulated in this simulation study. The first factor is the data distribution, including 1) multivariate normality and 2) multivariate nonnormality. The second factor is the sample size, including 1) 200, 2) 400, and 3) 600. In total, there are 6 combinations of data distribution and sample size. At each combination, we use the following population covariance matrix to generate 1000 random data sets:

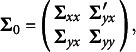

 where the first eight variables are the predictor variables and the last eight variables are the criterion variables^6^. The submatrices of **Σ**_0_ are

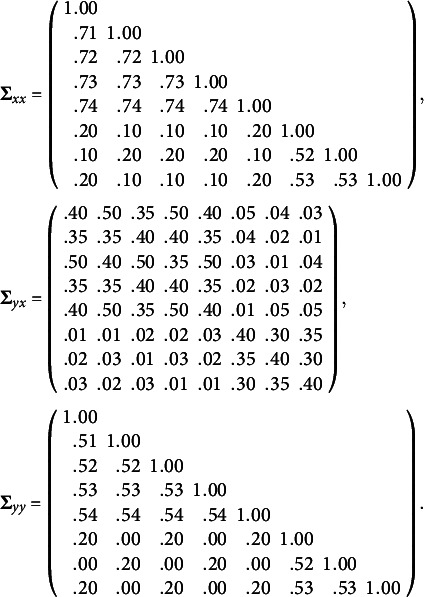



To generate the multivariate normal data, the RANDNORMAL function in SAS PROC IML is used. To generate the multivariate non-normal data, we use the procedure developed by Qu et al. (2020). This procedure is implemented by the MNONR package in R, which requires the user to specify the population values of multivariate skewness and multivariate kurtosis. In this simulation study, we set the values of multivariate skewness and multivariate kurtosis to 10 and 400, respectively^7^.

### 4.2 Data analysis and evaluation criteria

By applying RA to **Σ**_0_, we obtain the population values of the unrotated redundancy loadings and unrotated cross-loadings:

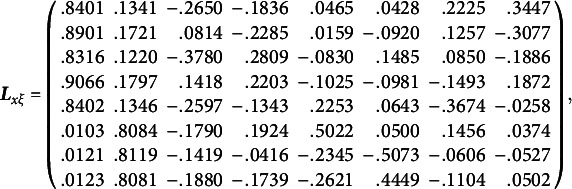




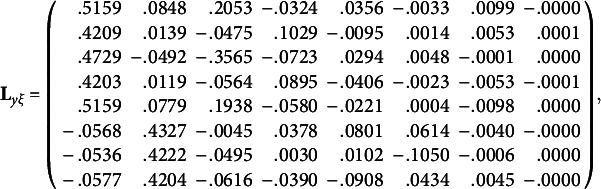

 and the first two population redundancy indices are .1399 and .0698, while the subsequent population redundancy indices are less than .03. Thus, for each random data set, we only rotate the first two columns of redundancy loadings. In terms of the rotation method, we use a widely accepted oblique rotation method: QUARTIMIN (Browne, 2001; Carroll, 1953) with Kaiser’s normalization (1958). In general, oblique rotations are more flexible than orthogonal rotations in the sense that oblique rotations can accommodate correlations among rotated factors/variates. If the rotated factors/variates are indeed uncorrelated, the resulting correlations from oblique rotations would be small and negligible. By applying QUARTIMIN to the first two columns of unrotated redundancy loadings, we obtain the population values of rotated redundancy loadings, rotated cross-loadings, and correlation of rotated redundancy variates:

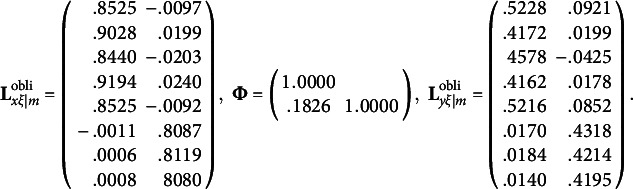

The normalized QUARTIMIN rotation is implemented by SAS PROC FACTOR, and the IJ method is implemented by customized code written in SAS PROC IML.

After the analyses are completed, we compute the means, standard deviations, and average standard error estimates across 1000 replications at each combination of data distribution and sample size. The standard deviations are used as the true standard errors to evaluate the performance of the IJ method. The first evaluation criterion we use is the relative bias of the average standard error estimate, which is calculated as

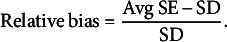

According to Hoogland and Boomsma (1998), the standard error estimate is acceptable when the absolute value of relative bias is less than .1. Additionally, we use the estimate and the associated standard error estimate to construct a symmetric 95% confidence interval (CI) and evaluate if the population value is included in the symmetric 95% CI. Thus, the second evaluation criterion is the coverage rate for each parameter across 1000 replications.

**Table 1:** Results from simulations under multivariate normality

Parm	*N* = 200				*N* = 400				*N* = 600			
	SD	Avg SE	Relative Bias	Coverage Rate (%)	SD	Avg SE	Relative Bias	Coverage Rate (%)	SD	Avg SE	Relative Bias	Coverage Rate (%)
*lx*_11_	.0604	.0603	−.0008	95.20	.0421	.0413	−.0190	94.80	.0315	.0332	.0530	96.60
*lx*_21_	.0471	.0492	.0447	95.70	.0319	.0325	.0207	95.80	.0258	.0258	.0018	95.50
*lx*_31_	.0657	.0653	−.0060	94.70	.0440	.0440	−.0008	95.30	.0360	.0355	−.0144	95.00
*lx*_41_	.0442	.0462	.0453	95.90	.0282	.0306	.0821	96.50	.0241	.0241	−.0019	95.30
*lx*_51_	.0616	.0604	−.0203	95.10	.0399	.0406	.0180	95.40	.0323	.0330	.0229	95.60
*lx*_61_	.0680	.0682	.0035	95.70	.0482	.0478	−.0097	95.00	.0395	.0390	−.0114	94.80
*lx*_71_	.0666	.0677	.0177	95.20	.0476	.0478	.0045	96.20	.0396	.0390	−.0140	94.20
*lx*_81_	.0672	.0674	.0034	94.90	.0472	.0476	.0085	95.40	.0371	.0387	.0424	96.20
*lx*_12_	.0680	.0681	.0021	94.50	.0475	.0482	.0147	93.60	.0389	.0394	.0124	94.70
*lx*_22_	.0730	.0752	.0291	94.80	.0549	.0534	−.0269	93.80	.0466	.0440	−.0549	93.40
*lx*_32_	.0786	.0792	.0075	94.00	.0531	.0552	.0391	94.70	.0441	.0454	.0292	94.60
*lx*_42_	.0797	.0802	.0067	93.40	.0589	.0573	−.0268	93.20	.0492	.0475	−.0344	93.60
*lx*_52_	.0627	.0656	.0463	95.40	.0458	.0457	−.0016	94.40	.0364	.0375	.0304	94.80
*lx*_62_	.0840	.0879	.0461	95.30	.0595	.0582	−.0228	94.00	.0469	.0474	.0107	96.00
*lx*_72_	.0813	.0849	.0434	94.70	.0575	.0567	−.0139	94.30	.0441	.0457	.0366	95.10
*lx*_82_	.0816	.0869	.0650	95.90	.0571	.0582	.0200	95.60	.0483	.0480	−.0055	93.80
*ϕ*_21_	.0942	.0937	−.0050	94.40	.0636	.0617	−.0292	94.90	.0509	.0501	−.0151	94.20
*ly*_11_	.0558	.0559	.0017	95.20	.0392	.0393	.0030	94.80	.0322	.0320	−.0065	96.60
*ly*_21_	.0580	.0594	.0238	95.70	.0415	.0423	.0194	95.80	.0348	.0344	−.0116	95.50
*ly*_31_	.0683	.0687	.0056	94.70	.0465	.0488	.0496	95.30	.0394	.0400	.0156	95.00
*ly*_41_	.0596	.0597	.0004	95.90	.0417	.0422	.0137	96.50	.0342	.0345	.0079	95.30
*ly*_51_	.0535	.0550	.0276	95.10	.0388	.0389	.0026	95.40	.0327	.0317	−.0296	95.60
*ly*_61_	.0764	.0758	−.0079	95.70	.0511	.0529	.0348	95.00	.0419	.0429	.0256	94.80
*ly*_71_	.0791	.0763	−.0362	95.20	.0535	.0532	−.0053	96.20	.0425	.0434	.0205	94.20
*ly*_81_	.0792	.0762	−.0379	94.90	.0514	.0534	.0393	95.40	.0430	.0436	.0153	96.20
*ly*_12_	.0870	.0894	.0276	94.50	.0611	.0610	−.0019	93.60	.0500	.0495	−.0091	94.70
*ly*_22_	.0755	.0790	.0472	94.80	.0545	.0544	−.0015	93.80	.0449	.0442	−.0167	93.40
*ly*_32_	.1136	.1152	.0140	94.00	.0803	.0807	.0057	94.70	.0669	.0667	−.0031	94.60
*ly*_42_	.0765	.0798	.0432	93.40	.0541	.0548	.0126	93.20	.0430	.0445	.0348	93.60
*ly*_52_	.0865	.0875	.0116	95.40	.0599	.0599	−.0002	94.40	.0498	.0487	−.0228	94.80
*ly*_62_	.0628	.0615	−.0208	95.30	.0422	.0420	−.0060	94.00	.0344	.0344	.0004	96.00
*ly*_72_	.0611	.0633	.0365	94.70	.0447	.0436	−.0244	94.30	.0365	.0356	−.0253	95.10
*ly*_82_	.0617	.0628	.0188	95.90	.0432	.0438	.0127	95.60	.0359	.0358	−.0014	93.80

*Note:* Parm = parameter, SD = standard deviation, Avg SE = average standard error, *lx* denotes the element of 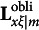 , *ϕ* denotes the element of **Φ**, *ly* denotes the element of 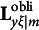 , and the subscript after *lx*, *ϕ*, and *ly* refers to the location of the element in the corresponding matrix.

**Table 2:** Results from simulations under multivariate nonnormality

Parm	*N* = 200				*N* = 400				*N* = 600			
	SD	Avg SE	Relative Bias	Coverage Rate (%)	SD	Avg SE	Relative Bias	Coverage Rate (%)	SD	Avg SE	Relative Bias	Coverage Rate (%)
*lx*_11_	.0678	.0662	−.0237	94.40	.0471	.0453	−.0391	94.00	.0364	.0369	.0157	94.80
*lx*_21_	.0482	.0509	.0558	97.00	.0324	.0336	.0379	95.80	.0255	.0265	.0404	96.50
*lx*_31_	.0689	.0691	.0039	95.40	.0479	.0474	−.0109	95.10	.0405	.0386	−.0461	93.90
*lx*_41_	.0452	.0489	.0835	97.40	.0310	.0311	.0025	94.60	.0242	.0248	.0233	95.80
*lx*_51_	.0655	.0647	−.0128	95.10	.0432	.0443	.0240	96.20	.0379	.0365	−.0389	94.90
*lx*_61_	.0681	.0696	.0226	96.00	.0489	.0489	−.0009	94.90	.0384	.0399	.0389	96.10
*lx*_71_	.0690	.0701	.0160	95.50	.0503	.0495	−.0150	95.10	.0405	.0406	.0012	94.70
*lx*_81_	.0655	.0679	.0377	95.30	.0494	.0473	−.0438	93.70	.0397	.0389	−.0212	93.70
*lx*_12_	.0660	.0683	.0347	95.00	.0476	.0484	.0164	95.50	.0375	.0396	.0568	95.50
*lx*_22_	.0766	.0741	−.0320	93.30	.0561	.0539	−.0375	92.60	.0459	.0443	−.0366	93.00
*lx*_32_	.0802	.0800	−.0023	93.80	.0544	.0565	.0370	95.70	.0453	.0458	.0107	95.20
*lx*_42_	.0840	.0810	−.0354	92.60	.0598	.0580	−.0298	93.30	.0491	.0478	−.0261	93.10
*lx*_52_	.0641	.0656	.0238	94.60	.0458	.0459	.0018	95.20	.0361	.0378	.0450	96.10
*lx*_62_	.0920	.0961	.0448	95.30	.0655	.0631	−.0369	93.60	.0528	.0519	−.0184	94.50
*lx*_72_	.0859	.0895	.0412	95.00	.0548	.0572	.0434	94.80	.0470	.0470	−.0004	95.20
*lx*_82_	.0891	.0886	−.0053	94.40	.0576	.0592	.0274	94.40	.0482	.0478	−.0074	95.10
*ϕ*_21_	.0954	.0973	.0201	95.40	.0672	.0642	−.0440	93.80	.0537	.0521	−.0308	94.70
*ly*_11_	.0693	.0641	−.0752	94.40	.0485	.0472	−.0269	94.00	.0388	.0387	−.0030	94.80
*ly*_21_	.0679	.0645	−.0491	97.00	.0486	.0476	−.0207	95.80	.0408	.0395	−.0318	96.50
*ly*_31_	.0804	.0785	−.0241	95.40	.0585	.0578	−.0120	95.10	.0476	.0479	.0056	93.90
*ly*_41_	.0676	.0646	−.0451	97.40	.0498	.0470	−.0554	94.60	.0396	.0388	−.0202	95.80
*ly*_51_	.0631	.0611	−.0327	95.10	.0438	.0444	.0129	96.20	.0368	.0362	−.0171	94.90
*ly*_61_	.0754	.0746	−.0105	96.00	.0526	.0527	.0011	94.90	.0431	.0429	−.0060	96.10
*ly*_71_	.0778	.0753	−.0315	95.50	.0525	.0528	.0055	95.10	.0445	.0432	−.0290	94.70
*ly*_81_	.0774	.0752	−.0276	95.30	.0550	.0532	−.0318	93.70	.0445	.0434	−.0253	93.70
*ly*_12_	.0895	.0916	.0245	95.00	.0610	.0632	.0370	95.50	.0512	.0512	.0003	95.50
*ly*_22_	.0813	.0814	.0018	93.30	.0559	.0556	−.0064	92.60	.0468	.0450	−.0383	93.00
*ly*_32_	.1188	.1196	.0071	93.80	.0857	.0847	−.0123	95.70	.0699	.0694	−.0076	95.20
*ly*_42_	.0811	.0817	.0074	92.60	.0560	.0558	−.0026	93.30	.0465	.0452	−.0269	93.10
*ly*_52_	.0875	.0907	.0360	94.60	.0633	.0624	−.0145	95.20	.0514	.0502	−.0228	96.10
*ly*_62_	.0696	.0683	−.0195	95.30	.0510	.0485	−.0488	93.60	.0418	.0399	−.0439	94.50
*ly*_72_	.0714	.0693	−.0304	95.00	.0468	.0480	.0276	94.80	.0417	.0396	−.0522	95.20
*ly*_82_	.0674	.0674	−.0005	94.40	.0472	.0469	−.0058	94.40	.0401	.0387	−.0353	95.10

*Note:* Parm = parameter, SD = standard deviation, SE = standard error, *lx* denotes the element of 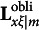 , *ϕ* denotes the element of **Φ**, *ly* denotes the element of 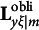 , and the subscript after *lx*, *ϕ*, and *ly* refers to the location of the element in the corresponding matrix.

### 4.3 Results

Because our purpose is to validate the standard error estimates from the IJ method, the means of rotated estimates are omitted in this section but can be found from the Supplementary Materials. Instead, we show the standard deviations, average standard errors, relative biases, and coverage rates in Tables 1 and 2 under multivariate normality and multivariate nonnormality, separately. It is observed that 1) the means are getting closer to their population values as the sample size increases, 2) all the absolute values of relative biases are less than 0.1, and 3) all the coverage rates are close to 95%. Therefore, we conclude that the IJ method performs well under both multivariate normality and multivariate nonnormality.

## 5 Two real examples

In this section, we use two real examples to demonstrate the interpretation of rotated redundancy variates. In the first example, the dimensionality was determined by a previous study, and we apply the normalized VARIMAX (Kaiser, 1958) for rotation. In the second example, we use the new criterion proposed by Gu et al. (2023) to determine the dimensionality and apply the normalized QUARTIMIN (Browne, 2001; Carroll, 1953) for rotation. The data and code for Example 1 can be found from the Supplementary Materials, and those for Example 2 can be requested from the first author.

### 5.1 Example 1

In the first example, we use the data from van Dam and van Trijp (2011), who collected 851 survey responses from the light users of sustainable products and applied RA to predict 10 variables measuring the motivational structure of sustainability by 15 variables that include psychographic variables and purchase behavior. The 10 motivational structure variables are *healthiness* (*y*_1_), *price* (*y*_2_), *convenience* (*y*_3_), *naturalness* (*y*_4_), *taste* (*y*_5_), *local production* (*y*_6_), *environment friendliness* (*y*_7_), *fair trade* (*y*_8_), *animal friendliness* (*y*_9_), and *waste* (*y*_10_). The 15 predictor variables are *concern for future consequences* (*x*_1_), *prevention focus* (*x*_2_), *promotion focus* (*x*_3_), *altruistic value* (*x*_4_), *biospheric value* (*x*_5_), *egoistic value* (*x*_6_), *NEP* ^8^ *scale* (*x*_7_), *connectedness to nature* (*x*_8_), *environment affect* (*x*_9_), *ethical orientation* (*x*_10_), *health prevention* (*x*_11_), *health promotion* (*x*_12_), *social SVO* ^9^ (*x*_13_), *individual SVO* (*x*_14_), and *competitive SVO* (*x*_15_). More details of these variables can be found from van Dam and van Trijp (2011).

By applying RA, we find that the first three redundancy indices are .2503, .0357, and .0074, which are exactly the same as those reported by van Dam and van Trijp (2011, p. 736), and all subsequent redundancy indices are smaller than .005. According to van Dam and van Trijp (2011), the first two redundancy indices are meaningful, and the third and subsequent redundancy indices can be ignored. Thus, we focus on the first two columns of the redundancy loadings and the cross-loadings.

To obtain the standard error estimates for unrotated RA estimates, we fit the original RA-L model. The estimation method we use include maximum likelihood (ML), which requires the multivariate normality assumption of the data, and ML with the Satorra–Bentler correction (referred to as MLSB hereafter), which does not require any distribution assumptions of the data. Table 3 shows the first two columns of **L** *_xξ_* and **L** *_yξ_* and the associated standard error estimates from ML and MLSB, separately.

**Table 3:** The first two columns of unrotated redundancy loadings and unrotated cross-loadings and the associated standard error estimates from ML and MLSB

	Point estimate			SE from ML			SE from MLSB		
		*ξ*_1_	*ξ*_2_		*ξ*_1_	*ξ*_2_		*ξ*_1_	*ξ*_2_
First two columns of unrotated redundancy loadings in 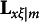	*x*_1_	−.0792	−.2800	*x*_1_	.0453	.0782	*x*_1_	.0502	.0924
	*x*_2_	.0038	−.0711	*x*_2_	.0448	.0771	*x*_2_	.0503	.0794
	*x*_3_	.0490	.1530	*x*_3_	.0447	.0729	*x*_3_	.0499	.0822
	*x*_4_	.6192	.4499	*x*_4_	.0326	.0525	*x*_4_	.0372	.0571
	*x*_5_	.8088	−.0372	*x*_5_	.0205	.0523	*x*_5_	.0238	.0561
	*x*_6_	−.0060	.2280	*x*_6_	.0452	.0754	*x*_6_	.0493	.0826
	*x*_7_	.5128	.0689	*x*_7_	.0354	.0711	*x*_7_	.0388	.0753
	*x*_8_	.7351	.0416	*x*_8_	.0250	.0564	*x*_8_	.0277	.0568
	*x*_9_	.5215	.0277	*x*_9_	.0349	.0667	*x*_9_	.0418	.0666
	*x*_10_	.7901	−.3497	*x*_10_	.0229	.0492	*x*_10_	.0257	.0558
	*x*_11_	.5938	.4012	*x*_11_	.0334	.0591	*x*_11_	.0382	.0644
	*x*_12_	.1680	−.0729	*x*_12_	.0440	.0792	*x*_12_	.0538	.0877
	*x*_13_	.1290	−.0224	*x*_13_	.0440	.0733	*x*_13_	.0449	.0718
	*x*_14_	−.1274	.0619	*x*_14_	.0440	.0732	*x*_14_	.0462	.0713
	*x*_15_	−.0136	.0573	*x*_15_	.0445	.0722	*x*_15_	.0436	.0758
		*ξ*_1_	*ξ*_2_		*ξ*_1_	*ξ*_2_		*ξ*_1_	*ξ*_2_
First two columns of unrotated cross-loadings in 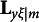	*y*_1_	.4749	.2177	*y*_1_	.0287	.0315	*y*_1_	.0329	.0351
	*y*_2_	.1132	.3267	*y*_2_	.0383	.0326	*y*_2_	.0462	.0341
	*y*_3_	−.0256	.2974	*y*_3_	.0385	.0357	*y*_3_	.0469	.0387
	*y*_4_	.6012	−.0103	*y*_4_	.0220	.0238	*y*_4_	.0262	.0294
	*y*_5_	.2069	.2904	*y*_5_	.0365	.0319	*y*_5_	.0447	.0329
	*y*_6_	.5684	−.0922	*y*_6_	.0235	.0271	*y*_6_	.0249	.0346
	*y*_7_	.6677	−.1256	*y*_7_	.0192	.0224	*y*_7_	.0209	.0282
	*y*_8_	.6591	−.0100	*y*_8_	.0196	.0274	*y*_8_	.0210	.0293
	*y*_9_	.5933	−.0063	*y*_9_	.0224	.0260	*y*_9_	.0252	.0265
	*y*_10_	.5522	−.0724	*y*_10_	.0241	.0269	*y*_10_	.0261	.0313

*Note:* SE = standard error estimate, ML = maximum likelihood, MLSB = maximum likelihood with the Satorra–Bentler correction.

By applying the normalized VARIMAX, we obtain 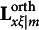 and 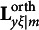 . To obtain the standard error estimates for rotated RA estimates, we fit the modified RA-L model for orthogonal rotations estimated by ULS, and apply the IJ method described in this paper. Table 4 shows 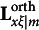 , 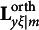 , and the associated standard error estimates from the IJ method.

**Table 4:** Rotated redundancy loadings, rotated cross-loadings, and the associated standard error estimates from the IJ method

	Point estimate			SE from IJ		
		*ξ*_1_	*ξ*_2_		*ξ*_1_	*ξ*_2_
Rotated redundancy loadings in 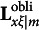	*x*_1_	−.0501	−.2867	*x*_1_	.0548	.0963
	*x*_2_	.0110	−.0703	*x*_2_	.0490	.0806
	*x*_3_	.0330	.1572	*x*_3_	.0475	.0846
	*x*_4_	.5698	.5110	*x*_4_	.0962	.1056
	*x*_5_	.**8084**	.0459	*x*_5_	.0258	.1306
	*x*_6_	−.0293	.2261	*x*_6_	.0514	.0826
	*x*_7_	.**5030**	.1211	*x*_7_	.0476	.0852
	*x*_8_	.**7269**	.1167	*x*_8_	.0345	.1268
	*x*_9_	.**5159**	.0810	*x*_9_	.0468	.0932
	*x*_10_	.**8217**	−.2669	*x*_10_	.0513	.1591
	*x*_11_	.5495	.4600	*x*_11_	.0888	.1164
	*x*_12_	.1746	−.0553	*x*_12_	.0519	.0914
	*x*_13_	.1306	−.0090	*x*_13_	.0438	.0689
	*x*_14_	−.1331	.0485	*x*_14_	.0462	.0698
	*x*_15_	−.0194	.0556	*x*_15_	.0432	.0765
		*ξ*_1_	*ξ*_2_		*ξ*_1_	*ξ*_2_
Rotated cross-loadings in 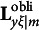	*y*_1_	.4501	.2652	*y*_1_	.0565	.0834
	*y*_2_	.0792	.3365	*y*_2_	.0750	.0364
	*y*_3_	−.0559	.2932	*y*_3_	.0636	.0387
	*y*_4_	.5991	.0513	*y*_4_	.0280	.1035
	*y*_5_	.1761	.3101	*y*_5_	.0666	.0404
	*y*_6_	.5749	−.0335	*y*_6_	.0258	.1076
	*y*_7_	.6770	−.0565	*y*_7_	.0212	.1111
	*y*_8_	.6566	.0576	*y*_8_	.0241	.1217
	*y*_9_	.5908	.0545	*y*_9_	.0276	.1014
	*y*_10_	.5567	−.0154	*y*_10_	.0259	.0942

*Note:* SE = standard error estimate, IJ = infinitesimal jackknife. The rotated redundancy loadings whose absolute values are significantly larger than .3 are in boldface.

Using the standard error estimates, we can test if the absolute value of a rotated redundancy loading in 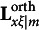 is larger than some cutoff value. Because the rotated redundancy loadings are correlations, we take .3 as the cutoff value, which means that at least 9% of the variance of a predictor variable must be shared with a rotated redundancy variate. Because we need to test the statistical significance of 30 rotated redundancy loadings simultaneously, it is necessary to adjust the typical significance level of .05. For convenience, we use the Bonferroni adjustment so that the adjusted significance level is .00167. It means that we will select a rotated redundancy loading if the associated *p*-value is smaller than .00167.

Based on the selected rotated redundancy loadings, we use the corresponding predictor variables to interpret the rotated redundancy variates. Specifically, the first rotated redundancy variate should be interpreted in terms of *biospheric value* (*x*_5_), *NEP scale* (*x*_7_), *connectedness to nature* (*x*_8_), *environment affect* (*x*_9_), and *ethical orientation* (*x*_10_); however, all the rotated redundancy loadings are smaller than .3 in the second column of 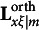 . Accordingly, the first rotated redundancy variate can be interpreted as people’s concern for environmental sustainability.

It is worth noting that if we only compared the absolute values of rotated redundancy variates against .3 but did not consider the sampling variability, we would select two more rotated redundancy loadings in the first column of 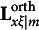 (i.e., .5698 and .5495) that correspond with *altruistic value* (*x*_4_) and *health prevention* (*x*_11_). Nevertheless, the significance tests indicate that the rotated redundancy loadings on these two variables are not really larger than .3, and their magnitude observed in this example just appears to be larger than .3 due to randomness. If these two variables would be used to interpret the first rotated redundancy variate, it would totally change the current interpretation of the first rotated redundancy variate. This reflects the advantage of the use of standard error estimates in selecting the rotated redundancy loadings.

**Table 5:** Results of the individual redundancy indices and cumulative redundancy for the real example

	Individual redundancy	Cumulative	95% CI for cumulative	95% CI for cumulative
	index	redundancy	redundancy from ML	redundancy from MLSB
1	.3116	.3116	(.2728, .3504)	(.2696, .3536)
2	.0458	.3574	(.3197, .3951)	(.3165, .3982)
3	.0341	.3915	(.3545, .4285)	(.3524, .4306)
4	.0086	.4001	(.3633, .4369)	(.3606, .4396)
5	.0062	.4063	(.3698, .4428)	(.3672, .4454)
6	.0017	.4080	(.3715, .4445)	(.3688, .4472)
7	.0002	.4081	(.3717, .4446)	(.3689, .4474)

*Note:* CI = confidence interval, ML = maximum likelihood, MLSB = maximum likelihood with the Satorra–Bentler correction.

### 5.2 Example 2

In the second example, we use the data from Jurukasemthawee et al. (2021) that collected responses from 424 young adults (mean age = 19.97, standard deviation of age = 1.64) on 9 psychological variables, serving as the predictor variables, and 7 spiritual well-being variables, serving as the outcome variables. The 9 predictor variables are *family and environment background* (*x*_1_), *crisis in life that contributed to self-development* (*x*_2_), *positive personal predisposition* (*x*_3_), *good role models* (*x*_4_), *faith activities* (*x*_5_), *mindfulness and self-regulation* (*x*_6_), *voluntary activities* (*x*_7_), *self-reflection* (*x*_8_), and *listening to positive experience* (*x*_9_). The 7 spiritual well-being variables are: *inner peace* (*y*_1_), *acceptance in diversity* (*y*_2_), *compassion* (*y*_3_), *self-transcendence* (*y*_4_), *value in self* (*y*_5_), *meaning in life* (*y*_6_), and *insight in learnings* (*y*_7_). Each of the predictor and outcome variables is computed from the sum of item scores that are measured on a Likert scale ranging from 0 to 6. The number of items used for each of the predictor and outcome variables is from 5 to 12 items. More details of these items can be found from Jurukasemthawee et al. (2021).

To determine the dimensionality in this example, we apply a new criterion proposed by Gu et al. (2023), which relies on the inferential information of redundancy indices. Specifically, we need to compare the lower limit of the 95% confidence interval (CI) for cumulative redundancy with some cutoff value. As a result, the smallest cumulative redundancy, of which the lower limit is larger than the specified cutoff value, can be identified. The identified cumulative redundancy determines the dimensionality in RA. In other words, we should retain the individual redundancy indices that constitute the identified cumulative redundancy. As for the cutoff value, we choose .3, meaning that at least 30% of the variance of criterion variables must be explained. To apply this new criterion, we need to fit the original RA-L model. As for the estimation method, we still use ML and MLSB.

Table 5 shows the results of the individual redundancy indices and cumulative redundancy for this example. By examining the lower limit of the 95% CI of cumulative redundancy, we find that the second cumulative redundancy is the smallest cumulative redundancy whose lower limit is larger than .3. It means that we should retain the first two individual redundancy indices. In addition, we notice that the second and third redundancy indices have comparable magnitude and both of them are distinctively larger than the fourth and subsequent redundancy indices, all of which are smaller than .01. Thus, we further study the difference between the second and third redundancy indices and their sum^10^. The results in Table 6 show that the 95% CI for the difference includes 0, indicating that the second and third redundancy indices are not significantly different; simultaneously, the lower limit of the 95% CI for their sum is larger than .06 and the upper limit is nearly .10, indicating that the second and third redundancy indices can explain about 6–10% of the variance of criterion variables. Based on these results, we decide to retain the first three redundancy variates. The unrotated redundancy loadings and unrotated cross-loadings of the first three redundancy variates are shown in Table 7.

**Table 6:** Difference between the 2nd and 3rd individual redundancy indices and sum of the 2nd and 3rd individual redundancy indices

	95% CI from ML	95% CI from MLSB
Difference = .0116	(−.0054, .0287)	(−.0059, .0292)
Sum = .0799	(.0630, .0968)	(.0615, .0983)

*Note:* Difference = the 2nd individual redundancy index − the 3rd individual redundancy index, Sum = the 2nd individual redundancy index + the 3rd individual redundancy index, CI = confidence interval, ML = maximum likelihood, MLSB = maximum likelihood with the Satorra–Bentler correction.

**Table 7:** The unrotated redundancy loadings and unrotated cross-loadings for the first three redundancy variates

	Point estimate				SE from ML				SE from MLSB			
		*ξ*_1_	*ξ*_2_	*ξ*_3_		*ξ*_1_	*ξ*_2_	*ξ*_3_		*ξ*_1_	*ξ*_2_	*ξ*_3_
First three columns of unrotated redundancy loadings in 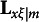	*x*_1_	.5734	−.1988	−.0432	*x*_1_	.0421	.0807	.1126	*x*_1_	.0479	.0894	.1433
	*x*_2_	.7946	−.2552	.1235	*x*_2_	.0268	.0784	.1129	*x*_2_	.0289	.0927	.1198
	*x*_3_	.9231	.1773	−.1194	*x*_3_	.0145	.0647	.0770	*x*_3_	.0150	.0756	.0878
	*x*_4_	.6062	.1196	.0278	*x*_4_	.0397	.0790	.0938	*x*_4_	.0444	.0921	.1097
	*x*_5_	.5404	.0012	.7650	*x*_5_	.0455	.3001	.0450	*x*_5_	.0483	.3251	.0500
	*x*_6_	.8144	−.3140	−.0987	*x*_6_	.0251	.0705	.1361	*x*_6_	.0271	.0925	.1558
	*x*_7_	.6313	.3061	.2998	*x*_7_	.0387	.1351	.1331	*x*_7_	.0396	.1710	.1501
	*x*_8_	.6755	.2733	−.2178	*x*_8_	.0360	.1084	.1222	*x*_8_	.0396	.1412	.1291
	*x_9_*	.6753	.2858	.2208	*x_9_*	.0358	.1053	.1213	*x_9_*	.0367	.1108	.1410
		*ξ*_1_	*ξ*_2_	*ξ*_3_		*ξ*_1_	*ξ*_2_	*ξ*_3_		*ξ*_1_	*ξ*_2_	*ξ*_3_
First three columns of unrotated cross-loadings in 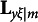	*y*_1_	.6652	−.3248	.0221	*y*_1_	.0289	.0353	.1164	*y*_1_	.0302	.0378	.1324
	*y*_2_	.4895	.1067	−.3511	*y*_2_	.0401	.1553	.0486	*y*_2_	.0488	.1823	.0502
	*y*_3_	.5849	.3443	−.0615	*y*_3_	.0350	.0448	.1135	*y*_3_	.0352	.0513	.1309
	*y*_4_	.5261	.0816	.0785	*y*_4_	.0356	.0504	.0471	*y*_4_	.0407	.0513	.0511
	*y*_5_	.2846	−.2592	−.2134	*y*_5_	.0491	.0991	.0887	*y*_5_	.0548	.0986	.0995
	*y*_6_	.6250	−.0803	.1263	*y*_6_	.0302	.0667	.0433	*y*_6_	.0333	.0809	.0455
	*y*_7_	.6393	.0681	.2091	*y*_7_	.0297	.0975	.0438	*y*_7_	.0337	.1185	.0460

*Note:* SE = standard error estimate, ML = maximum likelihood, MLSB = maximum likelihood with the Satorra–Bentler correction.

By applying the normalized QUARTIMIN, we obtain 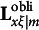 , 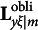 , and **Φ**. To obtain the standard error estimates for rotated RA estimates, we fit the modified RA-L model for oblique rotations estimated by ULS, and apply the IJ method described in this paper. Table 8 shows 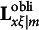 , 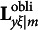 , and **Φ**, and the associated standard error estimates from the IJ method.

**Table 8:** Results of the rotated redundancy loadings, the rotated cross-loadings, and the correlations of the three rotated redundancy variates

	Point estimate				SE from IJ			
		*ξ*_1_	*ξ*_2_	*ξ*_3_		*ξ*_1_	*ξ*_2_	*ξ*_3_
Rotated redundancy loadings in 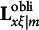	*x*_1_	.0327	.**5860**	−.0044	*x*_1_	.0506	.0412	.0802
	*x*_2_	.0280	.**7620**	.1847	*x*_2_	.0607	.0636	.0624
	*x*_3_	.**7291**	.3137	−.1069	*x*_3_	.0557	.0549	.0529
	*x*_4_	.4573	.1880	.0412	*x*_4_	.1072	.1065	.0719
	*x*_5_	.0870	.2054	.**8249**	*x*_5_	.0234	.0261	.0200
	*x*_6_	.0133	.**8776**	−.0424	*x*_6_	.0294	.0314	.0586
	*x*_7_	.**6529**	−.0719	.3102	*x*_7_	.0616	.0576	.0479
	*x*_8_	.**7570**	.0629	−.2290	*x*_8_	.0374	.0248	.0385
	*x_9_*	.**6671**	−.0106	.2309	*x_9_*	.0854	.0782	.0738
		*ξ*_1_	*ξ*_2_	*ξ*_3_		*ξ*_1_	*ξ*_2_	*ξ*_3_
Rotated cross-loadings in 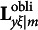	*y*_1_	.4762	.7345	.2226	*y*_1_	.0376	.0245	.0490
	*y*_2_	.4895	.4401	−.1660	*y*_2_	.0467	.0499	.0508
	*y*_3_	.6741	.4235	.1500	*y*_3_	.0284	.0423	.0472
	*y*_4_	.5142	.4545	.2501	*y*_4_	.0397	.0415	.0454
	*y*_5_	.1542	.3727	−.1209	*y*_5_	.0535	.0504	.0552
	*y*_6_	.5390	.6018	.3197	*y*_6_	.0377	.0320	.0463
	*y*_7_	.6126	.5559	.4097	*y*_7_	.0322	.0343	.0365
		*ξ*_1_	*ξ*_2_	*ξ*_3_		*ξ*_1_	*ξ*_2_	*ξ*_3_
Correlations between rotated redundancy variates in **Φ**	*ξ*_1_	1.0000			*ξ*_1_	N/A		
	*ξ*_2_	.7029	1.0000		*ξ*_2_	.0265	N/A	
	*ξ*_3_	.3218	.2234	1.0000	*ξ*_3_	.0345	.0289	N/A

*Note:* SE = standard error estimate, IJ = infinitesimal jackknife. The rotated redundancy loadings whose absolute values are significantly larger than .3 are in boldface.

Using the standard error estimates, we can test if the absolute value of a rotated redundancy loading in 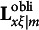 is larger than some cutoff value. Again, we take .3 as the cutoff value. Because we need to test the statistical significance of 27 rotated redundancy loadings simultaneously, it is necessary to adjust the typical significance level of .05. We use the Bonferroni adjustment again so that the adjusted significance level is .00185. It means that we will select a rotated redundancy loading if the associated *p*-value is smaller than .00185.

Based on the selected rotated redundancy loadings, we use the corresponding predictor variables to interpret the three rotated redundancy variates. Specifically, the first rotated redundancy variate should be interpreted in terms of *positive personal predisposition* (*x*_3_), *voluntary activities* (*x*_7_), *self-reflection* (*x*_8_), and *listening to positive experience* (*x*_9_); the second rotated redundancy variate should be interpreted in terms of *family and environment background* (*x*_1_), *crisis in life that contributed to self-development* (*x*_2_), and *Mindfulness and Self-Regulation* (*x*_6_); and the third rotated redundancy variate should be interpreted in terms of *faith activities* (*x*_5_). Accordingly, the first rotated redundancy variate can be interpreted as *positive personal predispositions that facilitated attention to positive experiences, self-reflection, and voluntary activities*; the second rotated redundancy variate can be interpreted as *safe family and environmental backgrounds that facilitated the use of mindfulness and self-regulation in transforming crisis into self-development*; and the third rotated redundancy variate can be interpreted as *engagement in activities that were related to own faiths*. Also, we found that the correlation between the first and second rotated redundancy variates is .7029 (with standard error estimate = .0265), suggesting that the first and second rotated redundancy variates share almost 50% of their variance. It implies that *positive personal predispositions* and *safe family and environmental backgrounds* are closely and significantly related. It should be noted that only oblique rotations can produce correlated rotated redundancy variates and the resulting correlations may bring more meaningful interpretations and insights to the study than the orthogonal rotations.

It is worth noting that if we only compared the absolute values of rotated redundancy variates against .3 but did not consider the sampling variability, we would select one more rotated redundancy loading in the third column of 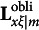 (i.e., .3102) that corresponds with *voluntary activities* (*x*_7_). Nevertheless, the significance test indicates that the rotated redundancy loading on this variable is not really larger than .3. If this variable would be used to interpret both the first and third rotated redundancy variates, it would cause some inconvenience in the interpretation, which in turn reflects the advantage of the use of standard error estimates in selecting the rotated redundancy loadings.

## 6 Discussions

In this paper, we specify two modified RA-L models for orthogonal and oblique rotations, separately, and describe the IJ method with the ULS fitting function to produce the standard error estimates for rotated RA estimates. Then, a simulation study is conducted to validate the performance of the IJ method. Additionally, two real examples are used to demonstrate the use of standard error estimates for rotated redundancy loadings when the rotated redundancy variates are interpreted. It was observed that the use of standard error estimates refines the selection of the rotated redundancy loadings and provides meaningful interpretations of the rotated redundancy variates in both examples.

Regarding the rotation method, one can use any of the rotation methods from the Crawford–Ferguson family (Crawford & Ferguson, 1970), while the choice of rotation method only changes one thing in the implementation of the IJ method. Specifically, the choice of rotation method determines the simplicity function (i.e., *h*^orth^ in equation 13 or *h*^obli^ in equation 14) used in the fourth type of constraints of the modified RA-L model, and the fourth type of constraints determines the last component of the Jacobian matrix (i.e., 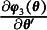 ) in equation (23). In other words, if a different rotation method is used, it is only the partial derivatives of the constraints in equation (13) or (14) that must be changed in the implementation of the IJ method.

Regarding the computation of partial derivatives, Lord (1975) and Browne and Du Toit (1992) recommended the use of numeric derivatives for nonstandard problems and models. Also, Jennrich (2008) reported good performance of numeric derivatives in the implementation of the IJ method. In our simulation study, we used numeric derivatives and obtained satisfactory results from the IJ method. Admittedly, one can argue that, in equations (23) and (24), the use of numeric derivatives is not as efficient/fast as the use of analytic derivatives. But this is a minor limitation in practical data analysis, because the difference in speed is trivial if there are only a few data sets to be analyzed. If there are a large number of data sets to be analyzed such as in simulation studies, then the difference would become noticeable. However, it is quite challenging to derive the necessary formulas for partial derivatives of different kinds of simplicity functions if the analytic derivatives must be used.

Finally, we would like to point out that the IJ method is a very general method for standard error estimation, but it is under-utilized in psychometrics. Historically, Jennrich and Clarkson (1980) first developed this method in the context of EFA. Later, Jennrich (2008) extended this method to the general framework of covariance structure analysis and referred to this method as the IJ method. Nonetheless, there are only two studies that applied the IJ method: Zhang et al. (2012) and Gu et al. (2021). We hope that our work would draw the attentions of not only the researchers but also the software developers who can develop accessible software programs to better promote the use of the IJ method.

## Supporting information

Gu et al. supplementary material Gu et al. supplementary material

## Footnotes

1 According to Gu et al. (2019), the names of the four models designed for CCA are 1) the COV-W model, 2) the COV-L model, 3) the CORR-W model, and 4) the CORR-L model. Each name has two parts that are separated by a dash. The first part is either COV or CORR. If the first part is COV, the model can analyze unstandardized variables (or a covariance matrix) and produce unstandardized estimates for the unique parameters. If the first part is CORR, the model can analyze not only unstandardized variables (or a covariance matrix) but also standardized variables (or a correlation matrix) and produce standardized estimates for the unique parameters. The second part of the name is either W or L, indicating the unique parameters subsumed by the model. If the second part is W, the model subsumes the weights as the unique parameters. If the second part is L, the model subsumes the loadings as the unique parameters.

2 In the EFA literature, there are two other methods that can be applied to compute the standard error estimates for rotated EFA estimates. The first method is the delta method (Archer & Jennrich, 1973; Jennrich, 1973), which requires a common factor model whose estimates are the unrotated EFA estimates. The second method is the augmented information matrix method (Jennrich, 1974), which requires a common factor model whose estimates are the rotated EFA estimates. In principle, these two methods can also be applied with the original and modified CORR-L models, separately, to produce the standard error estimates for rotated canonical loadings.

3 Gu et al. (2023) partially inherited the idea from Gu et al. (2019) to name the two models designed for RA. The first part of the name is always RA, rather than COV or CORR, because RA is defined to analyze standardized variables (or a correlation matrix) by Van Den Wollenberg (1977). The second part of the name is either W or L, indicating the unique parameters subsumed by the model. If the second part is W, the model subsumes the weights as the unique parameters. If the second part is L, the model subsumes the loadings as the unique parameters.

4 If possible, a subscript is used to indicate the order of a vector. For the null vector **0** on the right side of equation (3), it can be either **0**_*p*(*p* − 1)/2_ or **0**_*q*(*q* − 1)/2_, depending on the relative magnitude of *p* and *q*.

5
**Φ** is a correlation matrix due to the restriction imposed on **T**^obli^. That is,  .

6 By reviewing the RA literature, we found that most methodological articles often use a small number of predictor/criterion variables for illustrative purposes. For example, the artificial example used by Van Den Wollenberg (1977) has 4 predictor variables and 4 criterion variables, whereas Takane and Hwang (2005) set the minimum numbers of predictor and criterion variables to be 2 and 1, separately. As for the psychological examples analyzed by RA, the number of predictor/criterion variables can range from small to large. For example, Fornell (1979) used 14 predictor variables in the first example (i.e., Case One) but only 6 criterion variables in the second example (i.e., Case Two), while van Dam and van Trijp (2011) used RA to analyze 15 predictor variables and 10 criterion variables. Based on these findings, we choose to use 8 predictor variables and 8 criterion variables in our simulation study, which can be considered as a middle ground in the RA literature.

7 Qu et al. (2020) conducted a simulation study, where the number of variables is 2, 4, and 6, the values of multivariate skewness are 0, 1, 3, and 15, and the values of multivariate kurtosis are 10, 32, 61, and 91 (p. 943). They chose to report the results from three representative combinations of multivariate skewness and multivariate kurtosis, which were referred to as small, medium, and large nonnormality (p. 944). Qu et al. (2020) showed that both multivariate skewness and multivariate kurtosis are functions of the number of variables (equations 5 and 6) and that the value of multivariate kurtosis has a lower bound that depends on not only the number of variables but also the value of multivariate skewness (equations 17). Because we use 16 variables in this simulation study, which is about 3 times of the maximum number of variables (i.e., 6) used by Qu et al. (2020), we set the value of multivariate skewness to be 10, which is also about 3 times of multivariate skewness in medium nonnormality (i.e., 3) used by Qu et al. (2020). As for multivariate kurtosis, we decide to choose a number that is about 4 times of the maximum multivariate kurtosis (i.e., 91) used by Qu et al. (2020). Overall, the values we choose for multivariate skewness and multivariate kurtosis in our simulation study can be considered as a middle ground between medium and large nonnormality. The percentiles of multivariate skewness and multivariate kurtosis of the 16 variables and the percentiles of univariate skewness and univariate kurtosis of individual variables can be found from the Supplementary Materials of this paper.

8 NEP stands for New Ecological Paradigm. It is a scale to measure pro-environmental orientation.

9 SVO stands for social value orientation. It is a scale that allocates people based on the number of choices that maximize the own gain (individual), the joint gain (social), or the difference between own and other’s gain (competitive).

10 Gu et al. (2023) showed that individual redundancy indices are functions of the parameters of the original RA-L model. Thus, the difference between the second and third redundancy indices and their sum are also functions of the parameters of the original RA-L model. This allows us to apply the multivariate delta method to obtain the relevant inferential information.

## Supplementary material

To view supplementary material for this article, please visit http://doi.org/10.1017/psy.2024.8.
