# Supplementary material for: Infinitesimal Jackknife Estimates of Standard Errors for Rotated Estimates of Redundancy Analysis: Applications to Two Real Examples
Source: Psychometrika. 2025 Jan 3;90(1):183–207. doi: 10.1017/psy.2024.8 (PMC12478609; doi:10.1017/psy.2024.8)
Supplement: Gu et al. supplementary material [file S0033312324000085sup001.zip › Means of rotated estimates.docx]

| Parm | Population value | Mean of Rotated Estimates  under Multivariate Normality | | | Mean of Rotated Estimates  under Multivariate Nonnormality | | |
| --- | --- | --- | --- | --- | --- | --- | --- |
|  |  | *N* = 200 | *N* = 400 | *N* = 600 | *N* = 200 | *N* = 400 | *N* = 600 |
| *lx*_11_ | .8525 | .8232 | .8370 | .8428 | .8198 | .8383 | .8423 |
| *lx*_21_ | .9028 | .8718 | .8880 | .8931 | .8745 | .8881 | .8934 |
| *lx*_31_ | .8440 | .8137 | .8308 | .8347 | .8162 | .8283 | .8325 |
| *lx*_41_ | .9194 | .8907 | .9049 | .9098 | .8899 | .9060 | .9097 |
| *lx*_51_ | .8525 | .8224 | .8399 | .8430 | .8242 | .8403 | .8412 |
| *lx*_61_ | -.0011 | .0052 | .0005 | .0019 | .0076 | .0048 | .0024 |
| *lx*_71_ | .0006 | .0017 | .0034 | .0018 | .0044 | .0013 | .0027 |
| *lx*_81_ | .0008 | .0081 | .0061 | .0030 | .0046 | .0042 | .0016 |
| *lx*_12_ | -.0097 | -.0031 | -.0061 | -.0078 | -.0041 | -.0071 | -.0099 |
| *lx*_22_ | .0199 | .0234 | .0194 | .0219 | .0203 | .0196 | .0207 |
| *lx*_32_ | -.0203 | -.0138 | -.0156 | -.0170 | -.0126 | -.0125 | -.0180 |
| *lx*_42_ | .0240 | .0248 | .0215 | .0255 | .0265 | .0237 | .0265 |
| *lx*_52_ | -.0092 | -.0050 | -.0019 | -.0085 | -.0029 | -.0060 | -.0052 |
| *lx*_62_ | .8087 | .7620 | .7882 | .7939 | .7615 | .7845 | .7891 |
| *lx*_72_ | .8119 | .7670 | .7879 | .7969 | .7635 | .7935 | .7967 |
| *lx*_82_ | .8080 | .7668 | .7885 | .7900 | .7619 | .7864 | .7949 |
| *ϕ*_21_ | .1826 | .1416 | .1674 | .1706 | .1470 | .1645 | .1685 |
| *ly*_11_ | .5228 | .5280 | .5225 | .5248 | .5302 | .5270 | .5258 |
| *ly*_21_ | .4172 | .4310 | .4240 | .4232 | .4335 | .4240 | .4206 |
| *ly*_31_ | .4578 | .4737 | .4666 | .4636 | .4721 | .4659 | .4621 |
| *ly*_41_ | .4162 | .4301 | .4218 | .4219 | .4332 | .4234 | .4229 |
| *ly*_51_ | .5216 | .5298 | .5231 | .5243 | .5303 | .5250 | .5253 |
| *ly*_61_ | .0170 | .0150 | .0163 | .0170 | .0173 | .0141 | .0154 |
| *ly*_71_ | .0184 | .0112 | .0169 | .0167 | .0162 | .0162 | .0169 |
| *ly*_81_ | .0140 | .0127 | .0139 | .0111 | .0126 | .0143 | .0129 |
| *ly*_12_ | .0921 | .0835 | .0889 | .0906 | .0842 | .0867 | .0896 |
| *ly*_22_ | .0199 | .0156 | .0222 | .0214 | .0173 | .0210 | .0177 |
| *ly*_32_ | -.0425 | -.0358 | -.0307 | -.0402 | -.0349 | -.0347 | -.0408 |
| *ly*_42_ | .0178 | .0134 | .0190 | .0181 | .0146 | .0190 | .0171 |
| *ly*_52_ | .0852 | .0779 | .0840 | .0832 | .0786 | .0795 | .0830 |
| *ly*_62_ | .4318 | .4449 | .4414 | .4352 | .4487 | .4403 | .4384 |
| *ly*_72_ | .4214 | .4353 | .4300 | .4265 | .4362 | .4311 | .4265 |
| *ly*_82_ | .4195 | .4339 | .4287 | .4244 | .4369 | .4287 | .4240 |
