# Supplementary material for: Infinitesimal Jackknife Estimates of Standard Errors for Rotated Estimates of Redundancy Analysis: Applications to Two Real Examples
Source: Psychometrika. 2025 Jan 3;90(1):183–207. doi: 10.1017/psy.2024.8 (PMC12478609; doi:10.1017/psy.2024.8)
Supplement: Gu et al. supplementary material [file S0033312324000085sup001.zip › Modified RA_L_IJ.rtf]

Rotated redundancy loadings	

Lx_rotated		Lx_rotated_se		
-0.0501	-0.2867		0.0548	0.0963	
0.0110	-0.0703		0.0490	0.0806	
0.0330	0.1572		0.0475	0.0846	
0.5698	0.5110		0.0962	0.1056	
0.8084	0.0459		0.0258	0.1306	
-0.0293	0.2261		0.0514	0.0826	
0.5030	0.1211		0.0476	0.0852	
0.7269	0.1167		0.0345	0.1268	
0.5159	0.0810		0.0468	0.0932	
0.8217	-0.2669		0.0513	0.1591	
0.5495	0.4600		0.0888	0.1164	
0.1746	-0.0553		0.0519	0.0914	
0.1306	-0.0090		0.0438	0.0689	
-0.1331	0.0485		0.0462	0.0698	
-0.0194	0.0556		0.0432	0.0765	

A small p-value means that the rotated loading is larger than 0.3	

Lx_rotated_t		Lx_rotated_p		
-4.5568	-0.1383		1.0000	0.5550	
-5.8994	-2.8500		1.0000	0.9978	
-5.6209	-1.6881		1.0000	0.9543	
2.8055	1.9991		0.0025	0.0228	
19.7258	-1.9462		0.0000	0.9742	
-5.2662	-0.8943		1.0000	0.8144	
4.2631	-2.0987		0.0000	0.9821	
12.3630	-1.4453		0.0000	0.9258	
4.6172	-2.3484		0.0000	0.9906	
10.1664	-0.2080		0.0000	0.5824	
2.8086	1.3740		0.0025	0.0847	
-2.4138	-2.6765		0.9921	0.9963	
-3.8650	-4.2239		0.9999	1.0000	
-3.6090	-3.6038		0.9998	0.9998	
-6.4923	-3.1960		1.0000	0.9993	

Rotated cross-loadings	

Ly_rotated		Ly_rotated_se		
0.4501	0.2652		0.0565	0.0834	
0.0792	0.3365		0.0750	0.0364	
-0.0559	0.2932		0.0636	0.0387	
0.5991	0.0513		0.0280	0.1035	
0.1761	0.3101		0.0666	0.0404	
0.5749	-0.0335		0.0258	0.1076	
0.6770	-0.0565		0.0212	0.1111	
0.6566	0.0576		0.0241	0.1217	
0.5908	0.0545		0.0276	0.1014	
0.5567	-0.0154		0.0259	0.0942	

A small p-value means that the rotated cross-loading is larger than 0.3	

Ly_rotated_t		Ly_rotated_p		
2.6545	-0.4171		0.0040	0.6617	
-2.9435	1.0046		0.9984	0.1575	
-3.8377	-0.1765		0.9999	0.5700	
10.6829	-2.4029		0.0000	0.9919	
-1.8593	0.2497		0.9685	0.4014	
10.6687	-2.4761		0.0000	0.9934	
17.8242	-2.1912		0.0000	0.9858	
14.8126	-1.9926		0.0000	0.9768	
10.5303	-2.4214		0.0000	0.9923	
9.8954	-3.0218		0.0000	0.9987	


loading	
-0.0501	-0.2867	0.6474	-0.0458	0.2479	0.1235	0.1418	0.0818	0.2694	0.1117	
0.0110	-0.0703	-0.3894	0.2349	0.2586	0.2864	-0.6178	-0.0221	0.2298	0.2198	
0.0330	0.1572	-0.1514	0.1605	0.3287	0.3876	0.1646	-0.2631	0.0600	0.1750	
0.5698	0.5110	0.0089	0.0016	-0.3376	-0.0435	0.0677	0.0041	0.1913	0.3363	
0.8084	0.0459	0.1101	0.2962	0.0168	0.0198	0.1068	-0.0160	0.0354	0.2558	
-0.0293	0.2261	-0.3669	-0.1486	0.5158	0.2062	0.2289	0.4180	0.0830	0.3202	
0.5030	0.1211	0.3471	0.5028	-0.1375	0.1193	-0.1813	0.4442	0.0942	-0.1434	
0.7269	0.1167	-0.0748	0.1977	0.2995	-0.2608	0.0894	0.0520	-0.2814	-0.1908	
0.5159	0.0810	0.0290	0.4334	0.1112	-0.2119	-0.1340	0.0312	0.2820	0.1761	
0.8217	-0.2669	-0.2048	-0.3068	-0.0470	0.1932	0.0507	0.0412	0.0842	-0.0313	
0.5495	0.4600	0.2451	-0.2743	0.4293	0.1744	-0.2334	-0.1464	0.0791	-0.0715	
0.1746	-0.0553	0.5473	-0.3208	-0.0850	-0.2673	-0.2941	0.1237	-0.3445	0.3696	
0.1306	-0.0090	0.2126	-0.1300	-0.2049	-0.1254	0.2278	0.2455	0.4060	-0.0006	
-0.1331	0.0485	-0.1387	0.1257	0.1038	0.5388	0.0392	-0.0988	-0.5206	0.1213	
-0.0194	0.0556	0.0478	0.0912	0.1194	-0.1957	-0.1031	0.0197	-0.2927	0.2233	

loading	
0.3422	0.1498	-0.1789	0.3274	0.1636	
0.0692	0.3829	0.0480	-0.0451	-0.0130	
-0.1307	0.4598	-0.4034	-0.3492	-0.1535	
0.3308	0.0927	-0.0233	0.1271	-0.0957	
0.2055	-0.1680	0.1152	-0.0361	-0.2837	
0.0427	-0.0966	-0.2371	-0.1855	-0.2108	
-0.0661	-0.0582	-0.2000	-0.0489	0.1270	
0.1921	0.2742	0.0529	-0.0792	0.0904	
-0.2362	-0.1408	-0.0955	-0.0424	0.5151	
-0.1077	0.0194	-0.2080	0.0061	0.1015	
-0.1648	-0.1233	0.0533	-0.0527	0.0337	
-0.0107	-0.0131	-0.0346	-0.3343	0.1223	
-0.1813	0.4256	0.5820	-0.1734	-0.0576	
0.2283	-0.3588	-0.0989	0.0336	0.3938	
-0.4581	0.0555	-0.3147	0.6245	-0.2914	

0.0548	0.0963	0.0904	0.2696	0.1817	0.4797	0.4538	0.4029	0.2354	0.4711	0.5355	0.6614	0.3939	0.3544	0.8648	
0.0490	0.0806	0.1458	0.2271	0.2742	1.9143	0.9198	0.8294	0.3875	0.3735	0.5360	0.3214	0.4533	0.3860	0.6458	
0.0475	0.0846	0.1489	0.2169	0.1923	0.6008	1.2455	0.4357	0.3535	0.5540	0.7497	0.3708	0.4568	0.3975	0.6886	
0.0962	0.1056	0.1038	0.1836	0.0933	0.2651	0.2234	0.2384	0.1803	0.3082	0.3050	0.6676	0.4887	0.5065	0.7477	
0.0258	0.1306	0.1192	0.0771	0.1617	0.3694	0.1563	0.2082	0.1774	0.3455	0.6386	0.6057	0.3565	0.4053	0.2092	
0.0514	0.0826	0.1500	0.2985	0.1847	0.8581	0.8383	0.4209	0.4389	0.2856	0.4921	0.4736	0.3454	0.3793	0.2745	
0.0476	0.0852	0.1817	0.1699	0.2953	0.6742	0.6688	0.2781	0.3605	0.2634	0.2900	0.3116	0.2225	0.2586	0.2246	
0.0345	0.1268	0.1130	0.1799	0.1539	0.3591	0.8914	0.3601	0.1819	0.3184	0.5056	0.2314	0.3495	0.2751	0.5252	
0.0468	0.0932	0.1778	0.1284	0.2416	0.4792	0.6715	0.3582	0.2603	0.4268	0.6411	0.9358	0.4486	0.4642	0.4644	
0.0513	0.1591	0.1172	0.1002	0.1661	0.1883	0.6154	0.2477	0.1401	0.2423	0.2854	0.2734	0.1572	0.1919	0.1537	
0.0888	0.1164	0.1529	0.2348	0.1726	0.7656	0.6062	0.3291	0.2271	0.2146	0.2041	0.3141	0.2712	0.2602	0.4559	
0.0519	0.0914	0.1464	0.2195	0.2342	0.9068	0.9804	0.7116	0.3114	0.2938	0.4080	0.5000	0.4087	0.2926	0.1223	
0.0438	0.0689	0.1212	0.1766	0.1839	0.7389	0.5540	0.5349	0.3595	0.6476	0.7131	0.7020	0.2753	-0.1734	-0.0576	
0.0462	0.0698	0.1230	0.1769	0.2359	0.3097	1.6595	0.5695	0.3166	0.5577	0.6861	0.5263	-0.0989	0.0336	0.3938	
0.0432	0.0765	0.1327	0.1397	0.1909	0.3863	0.6803	0.4548	0.3122	0.7928	0.4379	0.0555	-0.3147	0.6245	-0.2914	

corr_y	
1.0000	0.2730	0.1317	0.4536	0.4426	0.3820	0.4426	0.4445	0.3983	0.4096	
0.2730	1.0000	0.2363	0.1208	0.3016	0.0766	0.1037	0.1282	0.1338	0.1314	
0.1317	0.2363	1.0000	0.0522	0.3288	-0.0229	-0.0225	0.0925	0.1102	-0.0421	
0.4536	0.1208	0.0522	1.0000	0.2530	0.5842	0.6057	0.5508	0.5282	0.4759	
0.4426	0.3016	0.3288	0.2530	1.0000	0.1601	0.2167	0.2310	0.2805	0.1741	
0.3820	0.0766	-0.0229	0.5842	0.1601	1.0000	0.5850	0.5435	0.4669	0.4934	
0.4426	0.1037	-0.0225	0.6057	0.2167	0.5850	1.0000	0.5976	0.5837	0.6251	
0.4445	0.1282	0.0925	0.5508	0.2310	0.5435	0.5976	1.0000	0.5675	0.4650	
0.3983	0.1338	0.1102	0.5282	0.2805	0.4669	0.5837	0.5675	1.0000	0.4481	
0.4096	0.1314	-0.0421	0.4759	0.1741	0.4934	0.6251	0.4650	0.4481	1.0000	

1.0000	0.0388	0.0402	0.0318	0.0359	0.0301	0.0312	0.0322	0.0334	0.0323	
0.0388	1.0000	0.0384	0.0384	0.0372	0.0392	0.0408	0.0388	0.0369	0.0381	
0.0402	0.0384	1.0000	0.0404	0.0334	0.0403	0.0389	0.0399	0.0380	0.0394	
0.0318	0.0384	0.0404	1.0000	0.0338	0.0262	0.0302	0.0299	0.0306	0.0346	
0.0359	0.0372	0.0334	0.0338	1.0000	0.0344	0.0346	0.0353	0.0342	0.0363	
0.0301	0.0392	0.0403	0.0262	0.0344	1.0000	0.0285	0.0290	0.0309	0.0327	
0.0312	0.0408	0.0389	0.0302	0.0346	0.0285	1.0000	0.0297	0.0284	0.0268	
0.0322	0.0388	0.0399	0.0299	0.0353	0.0290	0.0297	1.0000	0.0278	0.0349	
0.0334	0.0369	0.0380	0.0306	0.0342	0.0309	0.0284	0.0278	1.0000	0.0325	
0.0323	0.0381	0.0394	0.0346	0.0363	0.0327	0.0268	0.0349	0.0325	1.0000	

crossloading	
0.4501	0.2652	0.1328	-0.0988	0.0531	0.0218	-0.0443	-0.0198	-0.0009	-0.0009	
0.0792	0.3365	-0.0447	0.1128	-0.0207	-0.0195	-0.0539	0.0041	0.0203	0.0060	
-0.0559	0.2932	-0.1406	-0.0034	0.0692	0.0323	0.0454	-0.0240	-0.0059	-0.0043	
0.5991	0.0513	-0.0349	-0.0126	0.0410	0.0115	0.0009	0.0775	0.0170	-0.0080	
0.1761	0.3101	0.0869	-0.0263	-0.0571	-0.0461	0.0612	0.0202	-0.0096	0.0037	
0.5749	-0.0335	-0.1118	-0.0609	0.0309	-0.0384	-0.0118	0.0014	-0.0189	0.0180	
0.6770	-0.0565	0.0580	0.0540	0.0259	0.0151	0.0423	-0.0271	0.0423	0.0084	
0.6566	0.0576	-0.0932	-0.0632	-0.1050	-0.0008	-0.0090	-0.0271	0.0150	-0.0106	
0.5908	0.0545	0.0219	0.0726	-0.0463	0.0685	0.0007	0.0074	-0.0412	0.0040	
0.5567	-0.0154	0.0264	0.0800	0.0504	-0.0597	-0.0018	-0.0212	-0.0241	-0.0137	

0.0565	0.0834	0.0505	0.0520	0.0485	0.1465	0.0753	0.0509	0.0221	0.0040	
0.0750	0.0364	0.0545	0.0381	0.0563	0.1784	0.0602	0.0631	0.0200	0.0077	
0.0636	0.0387	0.0351	0.0589	0.0379	0.1519	0.1044	0.0594	0.0202	0.0058	
0.0280	0.1035	0.0283	0.0408	0.0347	0.0803	0.1177	0.0288	0.0485	0.0094	
0.0666	0.0404	0.0336	0.0474	0.0429	0.2003	0.1448	0.0694	0.0275	0.0058	
0.0258	0.1076	0.0347	0.0421	0.0413	0.0449	0.1158	0.0433	0.0232	0.0151	
0.0212	0.1111	0.0331	0.0345	0.0358	0.1371	0.0759	0.0764	0.0267	0.0108	
0.0241	0.1217	0.0436	0.0696	0.0367	0.0521	0.0587	0.0283	0.0218	0.0095	
0.0276	0.1014	0.0405	0.0455	0.0532	0.0352	0.1964	0.0564	0.0224	0.0077	
0.0259	0.0942	0.0473	0.0434	0.0437	0.0355	0.1806	0.0565	0.0268	0.0132	

std	
	COL1	COL2	COL3	COL4	COL5	COL6	COL7	COL8	COL9	COL10	COL11	COL12	COL13	
ROW1	0.8949	1.1177	1.2297	1.2000	1.3751	1.1980	0.7502	1.2657	1.0357	1.2337	1.0631	1.0775	0.4671	

std	
	COL14	COL15	COL16	COL17	COL18	COL19	COL20	COL21	COL22	COL23	COL24	COL25	
ROW1	0.3762	0.2041	1.0000	1.0000	1.0000	1.0000	1.0000	1.0000	1.0000	1.0000	1.0000	1.0000	

	COL1	COL2	COL3	COL4	COL5	COL6	COL7	COL8	COL9	COL10	COL11	COL12	COL13	
ROW1	0.0223	0.0283	0.0285	0.0280	0.0292	0.0295	0.0187	0.0297	0.0271	0.0293	0.0276	0.0266	0.0061	

	COL14	COL15	COL16	COL17	COL18	COL19	COL20	COL21	COL22	COL23	COL24	COL25	
ROW1	0.0113	0.0160	0.0262	0.0282	0.0230	0.0240	0.0313	0.0225	0.0245	0.0229	0.0220	0.0226	
