# Supplementary material for: Infinitesimal Jackknife Estimates of Standard Errors for Rotated Estimates of Redundancy Analysis: Applications to Two Real Examples
Source: Psychometrika. 2025 Jan 3;90(1):183–207. doi: 10.1017/psy.2024.8 (PMC12478609; doi:10.1017/psy.2024.8)
Supplement: Gu et al. supplementary material [file S0033312324000085sup001.zip › Original RA-L_ML.rtf]

Dx_hat	
	sd1	sd2	sd3	sd4	sd5	sd6	sd7	sd8	sd9	sd10	sd11	
ROW1	0.8949	1.1177	1.2297	1.2000	1.3751	1.1980	0.7502	1.2657	1.0357	1.2337	1.0631	

Dx_hat	
	sd12	sd13	sd14	sd15	
ROW1	1.0775	0.4671	0.3762	0.2041	

Dx_se	
0.0217	0.0271	0.0298	0.0291	0.0334	0.0291	0.0182	0.0307	0.0251	0.0299	0.0258	0.0261	0.0113	0.0091	0.0049	

Dy_hat	
sd16	sd17	sd18	sd19	sd20	sd21	sd22	sd23	sd24	sd25	
1.0000	1.0000	1.0000	1.0000	1.0000	1.0000	1.0000	1.0000	1.0000	1.0000	

Dy_se	
0.0243	0.0243	0.0243	0.0243	0.0243	0.0243	0.0243	0.0243	0.0243	0.0243	


Lx_hat	
	lx11	lx12	lx13	lx14	lx15	lx16	lx17	lx18	lx19	lx1_10	
ROW1	-0.0792	-0.2800	0.6474	-0.0458	0.2479	0.1235	0.1418	0.0818	0.2694	0.1117	
ROW2	0.0038	-0.0711	-0.3894	0.2349	0.2586	0.2864	-0.6178	-0.0221	0.2298	0.2198	
ROW3	0.0490	0.1530	-0.1514	0.1605	0.3287	0.3876	0.1646	-0.2631	0.0600	0.1750	
ROW4	0.6192	0.4499	0.0089	0.0016	-0.3376	-0.0435	0.0677	0.0041	0.1913	0.3363	
ROW5	0.8088	-0.0372	0.1101	0.2962	0.0168	0.0198	0.1068	-0.0160	0.0354	0.2558	
ROW6	-0.0060	0.2280	-0.3669	-0.1486	0.5158	0.2062	0.2289	0.4180	0.0830	0.3202	
ROW7	0.5128	0.0689	0.3471	0.5028	-0.1375	0.1193	-0.1813	0.4442	0.0942	-0.1434	
ROW8	0.7351	0.0416	-0.0748	0.1977	0.2995	-0.2608	0.0894	0.0520	-0.2814	-0.1908	
ROW9	0.5215	0.0277	0.0290	0.4334	0.1112	-0.2119	-0.1340	0.0312	0.2820	0.1761	
ROW10	0.7901	-0.3497	-0.2048	-0.3068	-0.0470	0.1932	0.0507	0.0412	0.0842	-0.0313	
ROW11	0.5938	0.4012	0.2451	-0.2743	0.4293	0.1744	-0.2334	-0.1464	0.0791	-0.0715	
ROW12	0.1680	-0.0729	0.5473	-0.3208	-0.0850	-0.2673	-0.2941	0.1237	-0.3445	0.3696	
ROW13	0.1290	-0.0224	0.2126	-0.1300	-0.2049	-0.1254	0.2278	0.2455	0.4060	-0.0006	
ROW14	-0.1274	0.0619	-0.1387	0.1257	0.1038	0.5388	0.0392	-0.0988	-0.5206	0.1213	
ROW15	-0.0136	0.0573	0.0478	0.0912	0.1194	-0.1957	-0.1031	0.0197	-0.2927	0.2233	

Lx_hat	
	lx1_11	lx1_12	lx1_13	lx1_14	lx1_15	
ROW1	0.3422	0.1498	-0.1789	0.3274	0.1636	
ROW2	0.0692	0.3829	0.0480	-0.0451	-0.0130	
ROW3	-0.1307	0.4598	-0.4034	-0.3492	-0.1535	
ROW4	0.3308	0.0927	-0.0233	0.1271	-0.0957	
ROW5	0.2055	-0.1680	0.1152	-0.0361	-0.2837	
ROW6	0.0427	-0.0966	-0.2371	-0.1855	-0.2108	
ROW7	-0.0661	-0.0582	-0.2000	-0.0489	0.1270	
ROW8	0.1921	0.2742	0.0529	-0.0792	0.0904	
ROW9	-0.2362	-0.1408	-0.0955	-0.0424	0.5151	
ROW10	-0.1077	0.0194	-0.2080	0.0061	0.1015	
ROW11	-0.1648	-0.1233	0.0533	-0.0527	0.0337	
ROW12	-0.0107	-0.0131	-0.0346	-0.3343	0.1223	
ROW13	-0.1813	0.4256	0.5820	-0.1734	-0.0576	
ROW14	0.2283	-0.3588	-0.0989	0.0336	0.3938	
ROW15	-0.4581	0.0555	-0.3147	0.6245	-0.2914	

Lx_se	
0.0453	0.0782	0.0885	0.2594	0.1717	0.4730	0.4356	0.3284	0.2107	0.4387	0.9454	
0.0448	0.0771	0.1396	0.2239	0.2472	1.8737	0.8854	0.7460	0.3457	0.3684	1.0946	
0.0447	0.0729	0.1302	0.2039	0.1966	0.5678	1.2190	0.4285	0.3520	0.5750	1.9162	
0.0326	0.0525	0.0980	0.1754	0.0906	0.2601	0.2252	0.2261	0.1842	0.3037	0.2914	
0.0205	0.0523	0.1116	0.0777	0.1571	0.3471	0.1484	0.1838	0.1692	0.3143	1.2284	
0.0452	0.0754	0.1411	0.2815	0.1726	0.7837	0.8168	0.3877	0.3803	0.3359	0.5271	
0.0354	0.0711	0.1808	0.1594	0.2668	0.6583	0.6405	0.2625	0.3525	0.2521	0.4786	
0.0250	0.0564	0.1095	0.1642	0.1482	0.3321	0.8214	0.3288	0.1870	0.3062	0.9544	
0.0349	0.0667	0.1653	0.1151	0.2301	0.4521	0.6779	0.3444	0.2301	0.4763	0.8999	
0.0229	0.0492	0.1134	0.0921	0.1601	0.1773	0.5973	0.2082	0.1326	0.2100	0.5959	
0.0334	0.0591	0.1397	0.2268	0.1742	0.7409	0.5664	0.3091	0.1921	0.2015	0.3762	
0.0440	0.0792	0.1342	0.2082	0.2388	0.9047	0.8552	0.5267	0.2776	0.3232	0.5500	
0.0440	0.0733	0.1170	0.1727	0.1735	0.7418	0.5445	0.4565	0.3120	0.6095	0.6995	
0.0440	0.0732	0.1213	0.1656	0.2250	0.2907	1.6424	0.5732	0.2913	0.5113	0.5761	
0.0445	0.0722	0.1105	0.1421	0.1583	0.3804	0.6536	0.3791	0.2913	0.6861	0.3864	

Lx_se	
1.2371	1.0120	0.8119	1.4051	
0.3276	0.6332	0.7665	0.9572	
1.1755	0.4915	1.1065	0.7650	
1.3792	0.8928	0.6267	1.2110	
1.1477	0.4053	0.5750	0.2783	
0.5847	0.3391	0.3574	0.2537	
0.6658	0.3271	0.2707	0.3308	
0.3560	0.5486	0.6130	0.7612	
1.7408	0.7601	0.4673	0.6848	
0.6360	0.2231	0.2952	0.1938	
0.6205	0.4975	0.4369	0.7116	
0.8885	0.5056	0.3459	999999.0	
0.7438	0.2938	999999.0	999999.0	
0.5030	999999.0	999999.0	999999.0	
999999.0	999999.0	999999.0	999999.0	


Psi_hat	
corr_y21	corr_y31	corr_y32	corr_y41	corr_y42	corr_y43	corr_y51	corr_y52	corr_y53	corr_y54	
1.0000	0.2730	0.1317	0.4536	0.4426	0.3820	0.4426	0.4445	0.3983	0.4096	
0.2730	1.0000	0.2363	0.1208	0.3016	0.0766	0.1037	0.1282	0.1338	0.1314	
0.1317	0.2363	1.0000	0.0522	0.3288	-0.0229	-0.0225	0.0925	0.1102	-0.0421	
0.4536	0.1208	0.0522	1.0000	0.2530	0.5842	0.6057	0.5508	0.5282	0.4759	
0.4426	0.3016	0.3288	0.2530	1.0000	0.1601	0.2167	0.2310	0.2805	0.1741	
0.3820	0.0766	-0.0229	0.5842	0.1601	1.0000	0.5850	0.5435	0.4669	0.4934	
0.4426	0.1037	-0.0225	0.6057	0.2167	0.5850	1.0000	0.5976	0.5837	0.6251	
0.4445	0.1282	0.0925	0.5508	0.2310	0.5435	0.5976	1.0000	0.5675	0.4650	
0.3983	0.1338	0.1102	0.5282	0.2805	0.4669	0.5837	0.5675	1.0000	0.4481	
0.4096	0.1314	-0.0421	0.4759	0.1741	0.4934	0.6251	0.4650	0.4481	1.0000	

Psi_se	
999999.0	0.0317	0.0337	0.0272	0.0276	0.0293	0.0276	0.0275	0.0289	0.0285	
0.0317	999999.0	0.0324	0.0338	0.0312	0.0341	0.0339	0.0337	0.0337	0.0337	
0.0337	0.0324	999999.0	0.0342	0.0306	0.0343	0.0343	0.0340	0.0339	0.0342	
0.0272	0.0338	0.0342	999999.0	0.0321	0.0226	0.0217	0.0239	0.0247	0.0265	
0.0276	0.0312	0.0306	0.0321	999999.0	0.0334	0.0327	0.0325	0.0316	0.0333	
0.0293	0.0341	0.0343	0.0226	0.0334	999999.0	0.0226	0.0242	0.0268	0.0260	
0.0276	0.0339	0.0343	0.0217	0.0327	0.0226	999999.0	0.0221	0.0226	0.0209	
0.0275	0.0337	0.0340	0.0239	0.0325	0.0242	0.0221	999999.0	0.0233	0.0269	
0.0289	0.0337	0.0339	0.0247	0.0316	0.0268	0.0226	0.0233	999999.0	0.0274	
0.0285	0.0337	0.0342	0.0265	0.0333	0.0260	0.0209	0.0269	0.0274	999999.0	


Ly_hat	
cly11	cly12	cly13	cly14	cly15	cly16	cly17	cly18	cly19	cly1_10	
0.4749	0.2177	0.1328	-0.0988	0.0531	0.0218	-0.0443	-0.0198	-0.0009	-0.0009	
0.1132	0.3267	-0.0447	0.1128	-0.0207	-0.0195	-0.0539	0.0041	0.0203	0.0060	
-0.0256	0.2974	-0.1406	-0.0034	0.0692	0.0323	0.0454	-0.0240	-0.0059	-0.0043	
0.6012	-0.0103	-0.0349	-0.0126	0.0410	0.0115	0.0009	0.0775	0.0170	-0.0080	
0.2069	0.2904	0.0869	-0.0263	-0.0571	-0.0461	0.0612	0.0202	-0.0096	0.0037	
0.5684	-0.0922	-0.1118	-0.0609	0.0309	-0.0384	-0.0118	0.0014	-0.0189	0.0180	
0.6677	-0.1256	0.0580	0.0540	0.0259	0.0151	0.0423	-0.0271	0.0423	0.0084	
0.6591	-0.0100	-0.0932	-0.0632	-0.1050	-0.0008	-0.0090	-0.0271	0.0150	-0.0106	
0.5933	-0.0063	0.0219	0.0726	-0.0463	0.0685	0.0007	0.0074	-0.0412	0.0040	
0.5522	-0.0724	0.0264	0.0800	0.0504	-0.0597	-0.0018	-0.0212	-0.0241	-0.0137	

Ly_se	
0.0287	0.0315	0.0479	0.0498	0.0478	0.1436	0.0710	0.0457	0.0183	0.0039	
0.0383	0.0326	0.0543	0.0358	0.0506	0.1748	0.0595	0.0560	0.0181	0.0066	
0.0385	0.0357	0.0341	0.0553	0.0346	0.1487	0.0984	0.0508	0.0209	0.0052	
0.0220	0.0238	0.0277	0.0363	0.0330	0.0754	0.1078	0.0268	0.0428	0.0093	
0.0365	0.0319	0.0333	0.0464	0.0387	0.1964	0.1374	0.0651	0.0235	0.0057	
0.0235	0.0271	0.0336	0.0409	0.0375	0.0453	0.1119	0.0370	0.0207	0.0142	
0.0192	0.0224	0.0304	0.0322	0.0351	0.1366	0.0632	0.0620	0.0263	0.0097	
0.0196	0.0274	0.0412	0.0641	0.0337	0.0486	0.0451	0.0242	0.0196	0.0090	
0.0224	0.0260	0.0404	0.0409	0.0476	0.0332	0.1976	0.0568	0.0207	0.0079	
0.0241	0.0269	0.0434	0.0407	0.0433	0.0330	0.1771	0.0487	0.0239	0.0117	
