# Supplementary material for: Infinitesimal Jackknife Estimates of Standard Errors for Rotated Estimates of Redundancy Analysis: Applications to Two Real Examples
Source: Psychometrika. 2025 Jan 3;90(1):183–207. doi: 10.1017/psy.2024.8 (PMC12478609; doi:10.1017/psy.2024.8)
Supplement: Gu et al. supplementary material [file S0033312324000085sup001.zip › Percentiles of mult_skew and mult_kurt.docx]

|  | | Multivariate Skewness | Multivariate Kurtosis |
| --- | --- | --- | --- |
| *N* = 200 | Min | 30.6955 | 310.8420 |
|  | 1^st^ | 36.7770 | 325.7830 |
|  | 5^th^ | 41.7796 | 333.1141 |
|  | 25^th^ | 46.7675 | 344.0171 |
|  | Median | 51.8368 | 354.5126 |
|  | 75^th^ | 59.0382 | 366.5907 |
|  | 95^th^ | 74.0054 | 390.2021 |
|  | 99^th^ | 93.8312 | 409.0606 |
|  | Max | 147.2119 | 455.1773 |
|  | | | |
| *N* = 400 | Min | 20.0999 | 332.8635 |
|  | 1^st^ | 24.1487 | 344.6443 |
|  | 5^th^ | 26.2715 | 349.5302 |
|  | 25^th^ | 30.6657 | 362.1798 |
|  | Median | 34.6400 | 371.7800 |
|  | 75^th^ | 40.4606 | 386.4361 |
|  | 95^th^ | 52.5165 | 412.9325 |
|  | 99^th^ | 64.6220 | 428.1890 |
|  | Max | 125.6794 | 502.3175 |
|  | | | |
| *N* = 600 | Min | 16.3990 | 340.9897 |
|  | 1^st^ | 18.8397 | 350.2503 |
|  | 5^th^ | 20.7793 | 357.4480 |
|  | 25^th^ | 24.5407 | 370.0544 |
|  | Median | 27.7047 | 379.5611 |
|  | 75^th^ | 32.1994 | 393.1660 |
|  | 95^th^ | 42.9333 | 417.0052 |
|  | 99^th^ | 53.8335 | 435.1561 |
|  | Max | 63.9079 | 477.9301 |
