# Supplementary material for: Infinitesimal Jackknife Estimates of Standard Errors for Rotated Estimates of Redundancy Analysis: Applications to Two Real Examples
Source: Psychometrika. 2025 Jan 3;90(1):183–207. doi: 10.1017/psy.2024.8 (PMC12478609; doi:10.1017/psy.2024.8)
Supplement: Gu et al. supplementary material [file S0033312324000085sup001.zip › Percentiles of univ_skew and univ_kurt.docx]

|  | | *X*_1_ | *X*_2_ | *X*_3_ | *X*_4_ | *X*_5_ | *X*_6_ | *X*_7_ | *X*_8_ |
| --- | --- | --- | --- | --- | --- | --- | --- | --- | --- |
| *N* = 200 | Min | -2.3519 | -2.2224 | -1.6097 | -1.1686 | -1.5111 | -3.0174 | -1.8767 | -1.3678 |
|  | 1^st^ | -1.4069 | -.5993 | -.9325 | -.8341 | -.6621 | -1.3767 | -.7494 | -.5813 |
|  | 5^th^ | -.6357 | -.2690 | -.2888 | -.2656 | -.2815 | -.5018 | -.2862 | -.2395 |
|  | 25^th^ | .1573 | .1571 | .1558 | .1686 | .1202 | .1639 | .1680 | .1351 |
|  | Median | .6457 | .4550 | .4216 | .4139 | .4017 | .5922 | .4572 | .4036 |
|  | 75^th^ | 1.1499 | .7931 | .7223 | .7031 | .7068 | 1.0301 | .7705 | .6728 |
|  | 95^th^ | 1.9743 | 1.3748 | 1.2312 | 1.1824 | 1.2785 | 2.0236 | 1.3095 | 1.1789 |
|  | 99^th^ | 3.1522 | 1.9499 | 1.9656 | 1.6742 | 1.9717 | 2.9067 | 1.8673 | 1.6996 |
|  | Max | 5.0234 | 3.5224 | 4.5694 | 2.6788 | 2.8719 | 5.9710 | 5.7603 | 3.3683 |
|  | | | | | | | | | |
| *N* = 400 | Min | -2.4878 | -1.5206 | -1.2061 | -1.3516 | -1.3735 | -3.4120 | -2.3056 | -.8505 |
|  | 1^st^ | -1.0517 | -.4873 | -.3718 | -.5253 | -.4424 | -1.3153 | -.7844 | -.3396 |
|  | 5^th^ | -.2339 | -.0865 | -.0752 | -.0701 | -.0860 | -.3318 | -.1568 | -.0636 |
|  | 25^th^ | .3068 | .2667 | .2495 | .2369 | .2323 | .3078 | .2573 | .2360 |
|  | Median | .7053 | .5397 | .4781 | .4644 | .4441 | .6307 | .4827 | .4219 |
|  | 75^th^ | 1.1077 | .7770 | .7165 | .6898 | .6665 | .9944 | .7313 | .6386 |
|  | 95^th^ | 1.9669 | 1.2882 | 1.1791 | 1.1784 | 1.1441 | 1.7335 | 1.2057 | 1.0945 |
|  | 99^th^ | 3.0543 | 1.8043 | 1.7105 | 1.7665 | 1.6858 | 2.8926 | 1.7928 | 1.4718 |
|  | Max | 4.5800 | 3.5137 | 2.3930 | 2.7663 | 2.5450 | 6.5601 | 3.9999 | 5.3887 |
|  | | | | | | | | | |
| *N* = 600 | Min | -1.3027 | -.5477 | -.5164 | -.7366 | -.6235 | -1.1919 | -.9502 | -.6351 |
|  | 1^st^ | -.5728 | -.2924 | -.2766 | -.3415 | -.2581 | -.6045 | -.2296 | -.2672 |
|  | 5^th^ | -.1304 | -.0068 | -.0207 | .0005 | -.0004 | -.1228 | .0027 | .0060 |
|  | 25^th^ | .3942 | .3226 | .2849 | .2742 | .2823 | .3910 | .3006 | .2692 |
|  | Median | .7061 | .5167 | .4958 | .4607 | .4629 | .6638 | .4971 | .4487 |
|  | 75^th^ | 1.0860 | .7416 | .7027 | .6610 | .6700 | .9844 | .7129 | .6438 |
|  | 95^th^ | 1.7924 | 1.1705 | 1.0612 | 1.0207 | 1.0356 | 1.6051 | 1.1471 | 1.0002 |
|  | 99^th^ | 2.4348 | 1.6295 | 1.5588 | 1.4399 | 1.4944 | 2.2272 | 1.7992 | 1.4173 |
|  | Max | 4.3598 | 2.0899 | 2.4280 | 2.1555 | 3.0208 | 3.2411 | 3.9186 | 2.6567 |

|  | | *Y*_1_ | *Y*_2_ | *Y*_3_ | *Y*_4_ | *Y*_5_ | *Y*_6_ | *Y*_7_ | *Y*_8_ |
| --- | --- | --- | --- | --- | --- | --- | --- | --- | --- |
| *N* = 200 | Min | -1.7397 | -3.8202 | -1.7977 | -1.1799 | -1.3360 | -1.9264 | -1.1483 | -.7460 |
|  | 1^st^ | -.9245 | -.8704 | -.5288 | -.7311 | -.4697 | -.7714 | -.5726 | -.4348 |
|  | 5^th^ | -.3932 | -.3550 | -.1929 | -.2378 | -.2224 | -.3372 | -.1903 | -.1631 |
|  | 25^th^ | .1248 | .1707 | .1301 | .0894 | .0914 | .1155 | .1136 | .0987 |
|  | Median | .4263 | .4433 | .3404 | .3778 | .2718 | .3754 | .3370 | .2897 |
|  | 75^th^ | .7400 | .7692 | .5586 | .6363 | .4823 | .7034 | .5753 | .5054 |
|  | 95^th^ | 1.3941 | 1.4067 | 1.0042 | 1.1871 | .8934 | 1.2419 | .9921 | .9042 |
|  | 99^th^ | 2.3176 | 2.2455 | 1.5659 | 2.2996 | 1.3039 | 1.8967 | 1.6104 | 1.3882 |
|  | Max | 7.8471 | 4.8730 | 2.9340 | 5.4828 | 1.9289 | 3.9238 | 2.3567 | 1.8621 |
|  | | | | | | | | | |
| *N* = 400 | Min | -3.4032 | -1.7032 | -.8264 | -1.3367 | -.8272 | -1.8873 | -.7946 | -.5466 |
|  | 1^st^ | -.6758 | -.5988 | -.2455 | -.4585 | -.3321 | -.5444 | -.3380 | -.2979 |
|  | 5^th^ | -.2012 | -.1552 | -.0414 | -.0985 | -.0919 | -.1572 | -.1004 | -.0717 |
|  | 25^th^ | .2203 | .2467 | .1875 | .2167 | .1631 | .1985 | .1758 | .1481 |
|  | Median | .4556 | .4829 | .3666 | .4298 | .3258 | .4248 | .3647 | .2919 |
|  | 75^th^ | .7191 | .7290 | .5556 | .6593 | .4894 | .6660 | .5521 | .4534 |
|  | 95^th^ | 1.2193 | 1.2554 | .8861 | 1.1324 | .8322 | 1.1336 | .8623 | .7647 |
|  | 99^th^ | 2.0920 | 1.9801 | 1.1888 | 1.7520 | 1.2297 | 1.7702 | 1.2963 | 1.0986 |
|  | Max | 3.0611 | 3.4391 | 2.1278 | 2.2268 | 1.8486 | 4.5509 | 2.1943 | 1.7915 |
|  | | | | | | | | | |
| *N* = 600 | Min | -1.0961 | -1.4865 | -.4242 | -.6333 | -.3616 | -.9993 | -.2693 | -.3406 |
|  | 1^st^ | -.4365 | -.4264 | -.1853 | -.2298 | -.1668 | -.3287 | -.1348 | -.1324 |
|  | 5^th^ | .0027 | -.0779 | .0158 | -.0171 | -.0022 | -.0286 | -.0036 | -.0074 |
|  | 25^th^ | .2968 | .2720 | .2326 | .2506 | .1893 | .2451 | .2249 | .1820 |
|  | Median | .4976 | .4915 | .3762 | .4171 | .3291 | .4582 | .3677 | .3187 |
|  | 75^th^ | .7198 | .7154 | .5344 | .5932 | .4706 | .6657 | .5335 | .4588 |
|  | 95^th^ | 1.1263 | 1.1285 | .8246 | .9575 | .7521 | 1.0780 | .7976 | .6869 |
|  | 99^th^ | 1.6017 | 1.8109 | 1.1009 | 1.3860 | 1.0719 | 1.5549 | 1.0364 | .8850 |
|  | Max | 2.1710 | 3.1335 | 2.5711 | 2.1248 | 1.6457 | 3.2263 | 1.7360 | 1.3972 |

|  | | *X*_1_ | *X*_2_ | *X*_3_ | *X*_4_ | *X*_5_ | *X*_6_ | *X*_7_ | *X*_8_ |
| --- | --- | --- | --- | --- | --- | --- | --- | --- | --- |
| *N* = 200 | Min | .2302 | -.1861 | -.0364 | -.2009 | -.1605 | -.1193 | -.2550 | -.2352 |
|  | 1^st^ | .9469 | .3252 | .1800 | .1013 | .1028 | .6388 | .1065 | -.0053 |
|  | 5^th^ | 1.5117 | .5779 | .5416 | .4162 | .4060 | 1.1442 | .5228 | .3623 |
|  | 25^th^ | 2.5965 | 1.3733 | 1.1479 | 1.0967 | 1.0485 | 2.1364 | 1.2484 | .9837 |
|  | Median | 4.0765 | 2.2003 | 1.8605 | 1.7705 | 1.7666 | 3.4563 | 2.0784 | 1.6735 |
|  | 75^th^ | 6.2495 | 3.4197 | 3.0102 | 2.8395 | 2.7692 | 5.6824 | 3.4369 | 2.6710 |
|  | 95^th^ | 12.9945 | 6.9567 | 6.4011 | 5.7690 | 6.4067 | 13.0907 | 6.8396 | 6.0659 |
|  | 99^th^ | 24.0498 | 12.2705 | 12.1984 | 9.4860 | 12.5315 | 25.1564 | 11.1200 | 1.3834 |
|  | Max | 46.4374 | 25.5898 | 42.5476 | 21.6324 | 25.4783 | 6.8902 | 62.1853 | 26.2626 |
|  | | | | | | | | | |
| *N* = 400 | Min | .4198 | .4704 | .1874 | .1013 | .2091 | 1.0175 | -.0655 | .2078 |
|  | 1^st^ | 1.3493 | .6759 | .5187 | .5328 | .4825 | 1.3394 | .5026 | .3517 |
|  | 5^th^ | 2.1454 | 1.0198 | .8780 | .7868 | .8249 | 1.7681 | .9576 | .6978 |
|  | 25^th^ | 3.3368 | 1.8357 | 1.5657 | 1.4562 | 1.3525 | 2.8461 | 1.7013 | 1.2556 |
|  | Median | 4.7632 | 2.6574 | 2.1904 | 2.0256 | 2.0455 | 4.0179 | 2.3597 | 1.8742 |
|  | 75^th^ | 7.0680 | 3.8412 | 3.3667 | 3.1500 | 3.0733 | 6.0595 | 3.5862 | 2.9184 |
|  | 95^th^ | 16.1324 | 7.8889 | 6.7907 | 7.0618 | 6.7758 | 12.9808 | 7.8071 | 5.4731 |
|  | 99^th^ | 29.3915 | 13.0478 | 11.6549 | 12.2413 | 11.5720 | 27.4997 | 17.5839 | 11.8057 |
|  | Max | 49.8651 | 29.4797 | 21.3784 | 24.1011 | 2.4447 | 78.5162 | 41.6972 | 69.1801 |
|  | | | | | | | | | |
| *N* = 600 | Min | 1.1812 | .3890 | .4781 | .3907 | .3112 | 1.1581 | .5083 | .1536 |
|  | 1^st^ | 1.8471 | .8723 | .8071 | .6606 | .6277 | 1.5635 | .7921 | .5705 |
|  | 5^th^ | 2.5253 | 1.2910 | 1.0889 | .9685 | .9521 | 2.1121 | 1.2251 | .8822 |
|  | 25^th^ | 3.8692 | 2.0674 | 1.7412 | 1.6108 | 1.6096 | 3.2588 | 1.9100 | 1.4874 |
|  | Median | 5.2412 | 2.7792 | 2.4507 | 2.1774 | 2.2351 | 4.4501 | 2.5292 | 2.1305 |
|  | 75^th^ | 7.4131 | 3.9308 | 3.3628 | 3.1689 | 3.2933 | 6.3430 | 3.6692 | 3.0668 |
|  | 95^th^ | 13.4421 | 6.8552 | 5.9909 | 6.0109 | 5.8692 | 11.7537 | 6.8378 | 5.7272 |
|  | 99^th^ | 23.1642 | 1.9834 | 12.7135 | 1.3800 | 1.0424 | 2.0171 | 13.5303 | 9.6735 |
|  | Max | 61.5589 | 16.8325 | 22.5697 | 2.0398 | 32.0632 | 36.8006 | 56.9747 | 27.0880 |

|  | | *Y*_1_ | *Y*_2_ | *Y*_3_ | *Y*_4_ | *Y*_5_ | *Y*_6_ | *Y*_7_ | *Y*_8_ |
| --- | --- | --- | --- | --- | --- | --- | --- | --- | --- |
| *N* = 200 | Min | -.1035 | -.1814 | -.3060 | -.2707 | -.3823 | -.2341 | -.6108 | -.5465 |
|  | 1^st^ | .2259 | .0526 | -.1322 | -.0314 | -.2110 | -.0133 | -.1942 | -.2783 |
|  | 5^th^ | .5414 | .4451 | .1400 | .2178 | -.0034 | .3091 | .1506 | -.0620 |
|  | 25^th^ | 1.1896 | 1.2411 | .6302 | .8351 | .4579 | 1.0197 | .6362 | .4575 |
|  | Median | 1.9653 | 1.9698 | 1.1836 | 1.4988 | .9307 | 1.7961 | 1.1721 | .9004 |
|  | 75^th^ | 3.4383 | 3.3361 | 2.0568 | 2.5679 | 1.5686 | 3.0031 | 2.0144 | 1.6438 |
|  | 95^th^ | 7.7865 | 7.5452 | 4.3358 | 5.9448 | 3.5571 | 6.6090 | 4.3783 | 3.5770 |
|  | 99^th^ | 14.9129 | 14.8421 | 8.4396 | 14.2601 | 6.6085 | 14.1299 | 1.8039 | 6.5701 |
|  | Max | 87.8393 | 44.8050 | 21.8441 | 53.6536 | 11.8918 | 35.7907 | 17.8393 | 12.0580 |
|  | | | | | | | | | |
| *N* = 400 | Min | .2833 | .3258 | -.0744 | -.0292 | -.2048 | .0615 | -.0199 | -.2349 |
|  | 1^st^ | .5589 | .5573 | .2379 | .3644 | .0764 | .3258 | .1327 | -.0038 |
|  | 5^th^ | .8872 | .9221 | .4786 | .6471 | .2883 | .7004 | .3795 | .1987 |
|  | 25^th^ | 1.6285 | 1.5954 | .9221 | 1.2300 | .7013 | 1.4208 | .9164 | .6314 |
|  | Median | 2.3972 | 2.3948 | 1.4248 | 1.8874 | 1.1741 | 2.0493 | 1.3391 | .9938 |
|  | 75^th^ | 3.6559 | 3.9298 | 2.2047 | 2.9506 | 1.8408 | 3.2129 | 1.9732 | 1.5734 |
|  | 95^th^ | 7.7382 | 7.7157 | 4.0396 | 6.1935 | 3.8475 | 7.1137 | 3.9948 | 3.4945 |
|  | 99^th^ | 17.3837 | 15.2458 | 6.5630 | 1.9873 | 7.4643 | 12.6291 | 8.4333 | 5.7692 |
|  | Max | 4.5162 | 34.1538 | 2.9861 | 22.8869 | 13.7202 | 45.1638 | 17.0411 | 15.9335 |
|  | | | | | | | | | |
| *N* = 600 | Min | .2513 | .4767 | .1643 | .0407 | .0369 | .3764 | .1357 | .0273 |
|  | 1^st^ | .7612 | .7040 | .4111 | .5140 | .2029 | .5520 | .3154 | .1765 |
|  | 5^th^ | 1.1539 | 1.1021 | .6505 | .7793 | .4261 | .8831 | .5789 | .3484 |
|  | 25^th^ | 1.8533 | 1.8160 | 1.0954 | 1.3502 | .8625 | 1.5424 | 1.0041 | .7679 |
|  | Median | 2.5761 | 2.6075 | 1.5699 | 1.9370 | 1.2223 | 2.2893 | 1.4552 | 1.1232 |
|  | 75^th^ | 3.7491 | 3.8373 | 2.2073 | 2.7731 | 1.8781 | 3.3841 | 2.1538 | 1.6757 |
|  | 95^th^ | 6.9193 | 7.2102 | 3.8500 | 5.3373 | 3.6200 | 6.6859 | 3.8819 | 3.1810 |
|  | 99^th^ | 12.5635 | 14.2100 | 6.3753 | 1.1148 | 5.9760 | 13.0389 | 6.9974 | 5.1297 |
|  | Max | 2.9656 | 33.9983 | 22.5865 | 16.8093 | 13.2473 | 36.5636 | 16.4561 | 1.2759 |
